# Synthesis, Anti-proliferative Activity, and Molecular Docking Study of New Series of 1,3-5-Triazine Schiff Base Derivatives

**DOI:** 10.3390/molecules25184065

**Published:** 2020-09-05

**Authors:** Hessa H. Al Rasheed, Azizah M. Malebari, Kholood A. Dahlous, Darren Fayne, Ayman El-Faham

**Affiliations:** 1 Department of Chemistry, College of Science, King Saud University P.O. Box 2455, Riyadh 11451, Saudi Arabia; kdahloos@ksu.edu.sa; 2 Department of Pharmaceutical Chemistry, College of Pharmacy, King Abdulaziz University, Jeddah 21589, Saudi Arabia; amelibary@kau.edu.sa; 3 Molecular Design Group, School of Biochemistry and Immunology, Trinity Biomedical Sciences Institute, Trinity College Dublin, Dublin 2, Ireland; fayned@tcd.ie; 4 Chemistry Department, Faculty of Science, Alexandria University, P.O. Box 426, Ibrahimia, Alexandria 12321, Egypt

**Keywords:** s-triazine, schiff base, MCF-7, HCT-116, apoptosis, molecular docking

## Abstract

Based on the use of *s*-triazine as a scaffold, we report here a new series of *s*-triazine Schiff base derivatives and their anti-proliferative activity against two cancer cell lines: human breast carcinoma (MCF-7), and colon cancer (HCT-116) compared with tamoxifen as a reference compound. Several derivatives exhibited growth inhibition activity in the sub-micromolar range. The results reveal that the *s*-triazine Schiff base derivatives showed varied activities and that the substituents on the *s*-triazine core have a great effect on the anti-proliferative activity. Compounds with a piperidino and benzylamino substituent on the *s*-triazine moiety **4b** and **4c** were most effective in both cell lines compared to the reference compound used. In addition, compound **4b** has a *para* chlorine atom on the benzylidine residue, demonstrating the most potent activity with IC_50_ values of 3.29 and 3.64 µM in MCF-7 and HCT-116, respectively. These results indicate that in general, the nature of the substituents on the triazine core and the type of substituent on the benzilyldene ring significantly influenced the anti-proliferative activity. The results obtained from the anti-proliferative activity and the molecular docking study indicate that *s*-triazine-hydrazone derivatives may be an excellent scaffold for the development of new anti-cancer agents.

## 1. Introduction

After cardiovascular diseases, cancer is the second most common cause of death globally, and its occurrence is predicted to increase dramatically in the near future. The high occurrence and death ratio of cancer is because there are more than 270 types of cancer that have a great tendency to resist chemotherapeutics and few cancers are diagnosed in their early stages. For all these reasons, research is essential for cancer treatments providing more effective and less toxic agents [1,2,3,4].

1,3,5-Triazine (s-triazine) is one of the most fascinating chemical cores in medicinal chemistry applications due to the broad range of biological activities such as anti-microbial [5], antifungal [6], antimalarial [7], carbonic anhydrase inhibitors [8,9,10,11,12] and anti-cancer agents [13,14,15,16,17]. 

On the other hand, Schiff bases are compounds formed by a condensation reaction between hydrazine or primary amines and carbonyl compounds to form azomethine type compounds (CH=NH-) and are considered to be important derivatives with biological, medicinal, clinical, pharmacological and analytical applications [18,19,20,21]. In addition, they are popular ligand precursors due to their versatility and ease of preparation. 

Schiff bases containing *s*-triazine core showed a wide diversity of biological activities such as anti-cancer, antimycobacterial, antibacterial, antidepressant, analgesic properties and enzyme inhibitors [22,23,24,25,26]. Moreover, 1,3,5-triazine derivatives have been shown to bind to and modulate estrogen receptor alpha (ERα) and estrogen receptor beta (ERβ) [27,28]. Very recently, Lu et al., reported the synthesis of 1,3,5-triazine-based derivatives as selective estrogen receptor degraders (SERDs). Most of these compounds showed anti-proliferative activities in MCF-7 cells [29].

**Figure 1:**
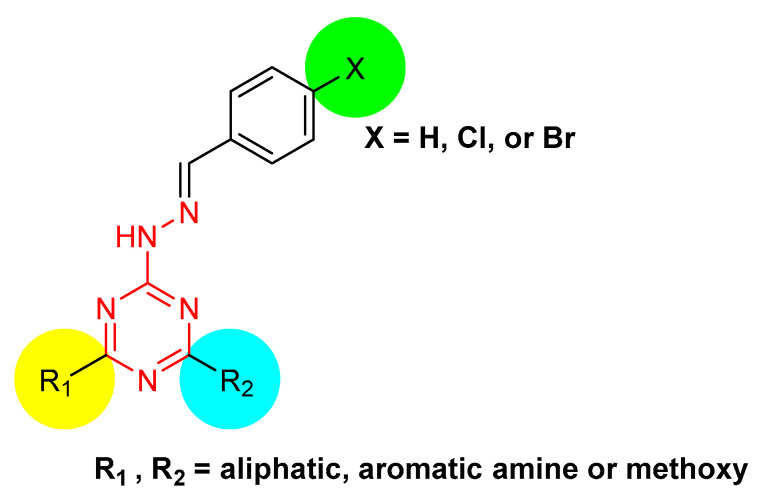
Structure of the novel synthesized *s*-triazine compounds.

In continuation of our studies on *s*-triazine-hydrazone derivatives and their anti-cancer activity against breast cancer cells (MCF-7) and colon cancer (HCT-116) [30,31,32], herein we report a new series of Schiff bases encompassing *s*-triazine core and benzylidene moieties with different substituents (Figure 1). The compounds’ anti-proliferative activities against breast cancer MCF-7 and colon carcinoma HCT-116 cell lines were evaluated. In addition, molecular docking of the compound series within the hypothesized biological target (ERα) will be discussed.

## 2. Results and Discussion

### 2.1. Chemistry 

The target compounds **4a-r** were synthesized as illustrated in Scheme 1 following the reported methods [31,32,33,34]. The hydrazine derivatives **2** were reacted with *p*-substituted benzaldehyde derivatives **3a-c** in the presence of acetic acid (AcOH) and using ethanol as a solvent to give the target compounds **4a-r** (Scheme 1). The structures of **4a-r** were established by elemental analysis, Fourier-transform infrared spectroscopy (FTIR), ^1^H-NMR, and ^13^C-NMR spectra (Supplementary information, Figures S1–S18).

### 2.2. Biology

#### 2.2.1. Anti-Proliferative Activity and Induced Apoptosis

*s*-Triazine-hydrazone derivatives **4a-r** were screened for their anti-proliferative activity against two representative human cancer cell lines; breast cancer MCF-7 and colon cancer HCT-116 and compared with tamoxifen [35] as a reference compound using (3-(4,5-dimethylthiazol-2-yl)-2,5-diphenyltetrazolium bromide (MTT) assay. Based on the data illustrated in Table 1 (Supporting information, Method S1), the *s*-triazine-hydrazone derivatives displayed varied anti-proliferative activity, with better efficacy on MCF-7 than HCT-116 cells. Generally, the nature of the substituents on the triazine ring significantly influenced the biological activity. *s*-Triazine-hydrazone derivatives **4m, 4n,** and **4o** (containing morpholine and phenylamine on the triazine core) showed potent anti-proliferative in MCF-7 and HCT-116 cells with IC_50_ values of 7.93, 6.10, and 6.58 µM and 5.10, 4.54, 10.41 µM, respectively. Interestingly, replacement of the morpholine ring in compounds **4m**–**o** with a methoxy group to furnished compounds **4p**–**r** maintained good to moderate anti-proliferative in MCF-7 with IC_50_ values in the range of 3.98–10.44 µM. To examine the significance of the phenylamino substituent on the triazine ring, a related series of benzylamino substituent analogs including compounds **4a**–**c** (containing piperidine on the triazine core) and compounds **4g**–**i** (containing morpholine on the triazine core) were next evaluated. Compounds **4b** and **4c,** with a piperidine ring, showed more potent anti-proliferative activity with, IC_50_ values of 3.29 and 4.63 µM in MCF-7 cell lines and 3.64 and 5.60 µM in HCT-116 cell lines, respectively compared to their corresponding analogs with a morpholine ring in compounds **4g**–**i** (IC_50_ values in range of 16.44–24.46 µM and 8.19–14.01 µM in MCF-7 and HCT-116, respectively) and compared with tamoxifen as a reference compound (IC_50_ values of 5.12 and 26.41 µM in MCF-7 and HCT-116 cells, respectively) as shown in Table 1. These results could reflect the importance of a piperidine substituent on the triazine ring as a less polar group for optimum activity in cancer cells. These results are consistent with our previously reported results, where the piperidine derivatives showed better activity compared to their corresponding morpholine analogs [33,34,36]. 

Conversely, replacement of the phenylamino substituent on the triazine moiety in compounds **4m**–**o** with a small group as a methoxy group (compounds **4d**–**f** and **4j**–**l**) significantly affected the anti-proliferative activity with a 2.5–5.5 and 3.7–12.5-fold loss in the potency on MCF-7 and HCT-116 cells, respectively. For example, replacement of phenylamino in morpholine substituted triazine derivative **4n** with a methoxy group in **4j** resulted in a marked decrease in the activity in both cell lines: IC_50_ values for **4n** were 6.10 and 4.54 µM vs. IC_50_ values for **4k** were 14.08 and 42.34 µM in MCF-7 and HCT-116 cells, respectively.

However, it was observed that methoxy *s*-triazine-hydrazone compounds **4p**–**r** with phenyl amine moiety, exhibited better anti-proliferative activity compared to their analogs **4d**–**f** (piperidine derivatives) and derivatives **4j**–**l** (morpholine derivatives) as shown in Table 1. For example, **4p** vs. **4d** and **4j**, exhibited significant activity against both tested cell lines. These results agreed with the reported data, where aromatic amines can help to enhance biological activity [37,38].

**Figure 2:**
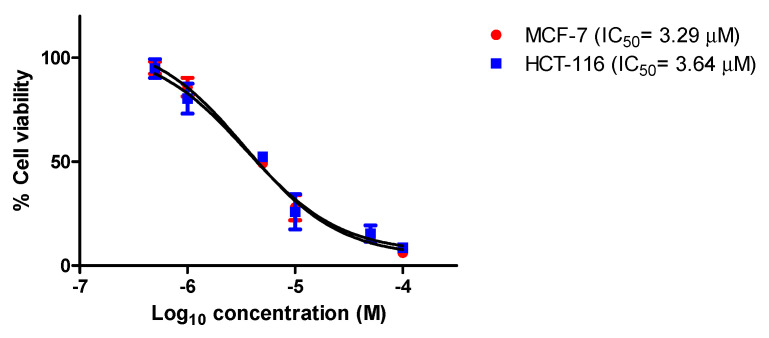
Anti-proliferative effect of **4b** in MCF-7 and HCT-116 cells. Cells were grown in 96-well plates and treated with **4b** at 0.1 µM to 100 µM 0.05–100 μM for 72 h. Cell viability was expressed as a percentage of vehicle control [ethanol 0.1% (v/v)] treated cells. The values represent the mean ± S.E.M. for three independent experiments performed in triplicate.

**Figure 3:**
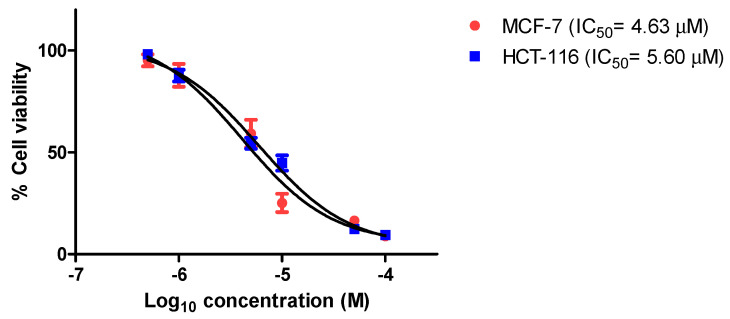
Anti-proliferative effect of **4c** in MCF-7 and HCT-116 cells. Cells were grown in 96-well plates and treated with **4c** at 0.1 µM to 100 µM for 72 h. Cell viability was expressed as a percentage of vehicle control [ethanol 0.1% (v/v)] treated cells. The values represent the mean ± S.E.M. for three independent experiments performed in triplicate.

With the exception of compounds **4d**–**f** and **4j**–**l**, the introduction of electron-withdrawing groups such as chloro and bromo substituent on the benzylidene ring with different substituted *s*-triazine motifs demonstrated good anti-cancer activities against both cell lines in the low micromolar range compared to its corresponding unsubstituted analogs. Derivatives with piperidino and benzylamino substituent on the triazine moiety **4b** (chloro substituent) and **4c** (bromo substituent) showed the most potent activities in this series in both cell lines as shown in Table 1 and Figure 2 and Figure 3.

Finally, *s*-triazine-hydrazone derivatives that encompass benzylamino and piperidino rings on the *s*-triazine core can be envisaged as interesting novel molecules with promising anti-proliferative activities in MCF-7 and HCT-116 cell lines. 

**Figure 4:**
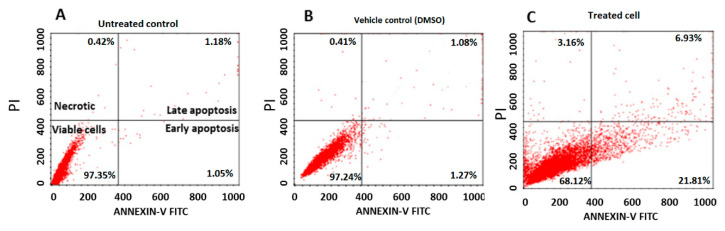
Flow cytometric analysis (Annexin V-FTIC/PI assay) of MCF-7 cells exposed for 24 h to **4b** (C) (approximately 2x IC_50_ value), respectively. A = untreated control, and B vehicle control (DMSO). The represented dot plots showing percentage of viable, early apoptotic, late apoptotic, and necrotic cells.

Based on the obtained results, we examined apoptosis in MCF-7 cells after treatment with the most active compound **4b**. The percentage of early and late apoptotic cells was determined by double staining with Annexin V and PI via flow cytometry. Positioning of quadrants on dot plots was designated, and living cells (Annexin V−/PI−), early apoptotic cells (Annexin V+/PI−), late apoptotic cells (Annexin V+/PI+) and necrotic cells (Annexin V−/PI+) were identified. The data shown in Figure 4 prove that the MCF-7 cells were incubated with **4b** at a concentration of 6 µM for 24 h decreased the population of viable cells and increasing the percentage of apoptotic cells. The percentage of early and late apoptotic cells together of **4b** increased after 24 h to 21.81% and 6.93%, respectively at 6 µM when compared to the control cells (2.23%) as shown in Figure 4.

These results show the ability of **4b** to induce apoptosis in cancer cells and may deliver an excellent scaffold for the development of anti-cancer drug candidates. 

#### 2.2.2. Molecular Docking Study 

Estrogen actions are mainly mediated through two nuclear estrogen receptor (ER) subtypes namely ERα and ERβ [41,42]. In Western blot analyses, the antibody against ERα detected a single sharp band related to the expected molecular weight of 72 kD in all MCF-7 subclones. In agreement with Western blot analyses, a high expression of the ERα mRNA was detected in MCF-7 [43]. Accordingly, an in-depth binding pose analysis on all compounds with ERα was performed. 

The top docked pose of each compound was analyzed and mapping to the X-ray structure ligands binding poses was considered (Supporting information, Method S2). The triazine derivatives **4a**–**r** were scored following docking, in order of preference. This ranking broadly maps to the cellular efficacy biological data, as the **4a**–**c** series are the most potent compounds while the **4j**–**l** and **4d**–**f** series are the least potent and **4p**–**q** have moderate potency.

**Figure 5:**
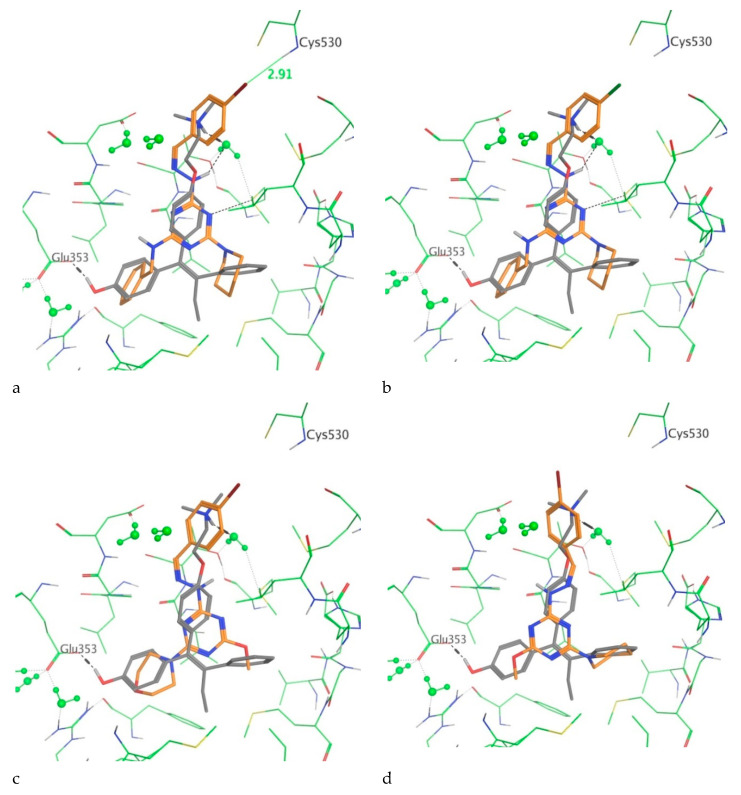
Best ranked docked poses of (**a**) **4c**, (**b**) **4b**, (**c**) **4l** and (**d**) **4f** in ERα overlaid on the 4-OHT X-ray structure 3ERT generated with the Molecular Operating Environment (MOE) 2019 software. Carbon atoms are illustrated in grey for 4-OHT, orange for the docked compounds and light green for the protein (oxygen atoms – red; nitrogen – dark blue; sulfur – yellow; bromine – dark red and chlorine – dark green). ERα and associated water molecules are in light green.

Analysis of the predicted binding poses assisted to illustrate the potency trends. The most potent compounds (**4b** and **4c**) had near identical scores (−7.8387 and −7.8355 respectively). As shown in Figure 5a and b, the halogenated benzylidene ring mapped to the tertiary amine group of 4-hydroxytamoxifen (4-OHT), the triazine ring mapped to the 4-OHT A ring, the benzyl ring mapped to the 4-OHT B ring and the piperidine ring mapped to the 4-OHT C ring. Binding was primarily driven by hydrophobic and van der Waals interactions due to the benzyl and piperidine rings occupying apolar sub-pockets in the ERα binding site. The benzyl group was in a sub-pocket comprising of Leu349, Ala350, Leu391, and Phe404. The group did not make interactions with the polar Glu353 and Arg394 amino acids deeper in the sub-pocket, which will be a future route for compound optimization. The piperidine ring occupied a sub-pocket encompassing Val418, Ile424, Leu525, and Leu346. The amine of the benzylidene chain can make a hydrogen bond donor interaction with a co-crystallized water molecule but did not make a hydrogen bond with the important Asp351 residue. The halogens are 2.91 Å from the backbone nitrogen atom of Cys530 so may make an attractive electrostatic interaction.

Conversely, the worst ranked docked series, for example, **4l** (Figure 5c) was unable to recapitulate the favorable interactions formed by the benzyl group of the **4a**–**c** series. The more polar morpholine ring in **4g**–**i** series is also not preferred to the piperidine ring of the **4a**–**c** series as it was in a hydrophobic sub-pocket. Due to the smaller substituent sizes, the docked compound was located lower in the binding site so the triazine group is not overlaid on the 4-OHT A ring. Similar binding poses were found for the piperidine and methoxy on the triazine series **4d**–**f** (i.e., **4f**, Figure 5d), in that the methoxy group occupies one of the hydrophobic sub-pockets and the compound is located lower in the binding site.

In summary, the results obtained from molecular docking are consistent with the results obtained from the anti-proliferative activity screening, where the most active series are compounds with piperidine and benzylamine on the triazine core.

## 3. Materials and Methods 

All reagents and solvents purchased from commercial suppliers and used without further purification. ^1^H-NMR and ^13^C-NMR spectra were recorded on a JEOL 400 MHz spectrometer (JEOL, Ltd., Tokyo, Japan), and chemical shift (*δ*) values were expressed in ppm. Elemental analyses performed on Perkin–Elmer 2400 elemental analyzer (PerkinElmer, Inc.940 Winter Street, Waltham, MA, USA). Melting points were recorded on a Mel-Temp apparatus Sigma-Aldrich Chemie GmbH, 82024 Taufkirchen, Germany) in an open capillary and are uncorrected. Fourier transform infrared spectroscopy (FTIR) recorded on Shimadzu 8201 PC FTIR spectrophotometer (Shimadzu, Ltd., Kyoto, Japan). The reaction was follow-up and checks of the purity using thin layer liquid chromatograph (TLC) on silica gel-protected aluminum sheets (Type 60 GF254, Merck).

### 3.1. General Method for the Synthesis of 1,3,5-triazine Schiff Base Derivatives

2-Hydrazino-4,6-disubstituted-1,3,5-triazine derivatives **2** were synthesized first following the reported methods [26,32,33,34,36] and used directly without further purification into the next step.

Hydrazine derivatives **2** were reacted with aldehyde derivatives **3a**–**c** (10 mmol) in ethanol (30 mL) in the presence of 2–3 drops of acetic acid at room temperature. The reaction mixture was refluxed for 4–6 h, and the progress of the reaction was follow by TLC using ethyl acetate-hexane 2:1. After completion of the reaction, the mixture left to cool down to room temperature and the solid product was collected by filtration, washed with cooled ethanol, and then dried at room temperature.

*N-benzyl-4-(2-benzylidenehydrazinyl)-6-(piperidin-1-yl)-1,3,5-triazin-2-amine* (**4a)**. the product was obtained as a white solid in yield 83%; mp 148–150 °C; IR (KBr, cm^−1^): 3241 (NH), 1662 (C=N), 1590, 1545 (C=C); ^1^H-NMR (400 MHz, DMSO-*d_6_*): δ = 1.40 (brs, 4H, 2CH_2_), 1.55 (brs, 2H, CH_2_), 3.65 (brs, 4H, 2 NCH_2_-), 4.39–4.45 (m, 2H, CH_2_-NH), 7.16–7.36 (m, 8H, Ar), 7.57 (s, 2H, Ar), 8.03 (s, 1H, CH), 10.57 (brs, 1H, NH) ), 10.64 (brs, 1H, NH) ppm; ^13^C-NMR (100 MHz, DMSO-*d_6_*): δ = 24.9, 25.9, 43.4, 56.6, 126.7, 126.9, 127.9, 128.6, 129.2, 129.4, 135.7, 141.3, 141.9, 164.8, 166.5 ppm. Anal. Calcd for C_22_H_25_N_7_ (387.48): C, 68.19; H, 6.50; N, 25.30. Found C, 68.32; H, 6.66; N, 25.50.

*N-benzyl-4-(2-(4-chlorobenzylidene)hydrazinyl)-6-(piperidin-1-yl)-1,3,5-triazin-2-amine* (**4b**). the product was obtained as a white solid in yield 88%; mp 200–202 °C; IR (KBr, cm^−1^): 3376 (NH), 1592 (C=N), 1516,1442 (C=C); ^1^H-NMR (400 MHz, DMSO-*d_6_*): δ = 1.40 (brs, 4H, 2CH_2_), 1.55 (brs, 2H, CH_2_), 3.64 (brs, 4H, 2 NCH_2_-), 4.38–4.45 (m, 2H, CH_2_-NH), 7.16 (t, 1H, *J* = 7.0 Hz, Ar), 7.23–7.29 (m, 4H, Ar), 7.42 (d, 2H, *J* = 8.5 Hz, Ar), 7.58 (d, 2H, *J* = 8.0 Hz, Ar), 8.01 (s, 1H, CH), 10.66 (brs, 1H, NH), 10.71 (s, 1H, NH) ppm; ^13^C-NMR (100 MHz, DMSO-*d_6_*): δ = 24.9, 25.9, 43.5, 56.6, 126.9, 127.9, 128.4, 128.6, 129.3, 133.7, 134.7, 140.5, 141.2, 164.6, 166.4 ppm. Anal. Calc. for C_22_H_24_ClN_7_ (421.93): C, 62.63; H, 5.73; N, 23.24. Found C, 62.54; H, 5.68; N, 23.45.

*N-benzyl-4-(2-(4-bromobenzylidene)hydrazinyl)-6-(piperidin-1-yl)-1,3,5-triazin-2-amine* (**4c**). the product was obtained as a white solid in yield 84%; mp 195–197 °C; IR (KBr, cm^−1^): 3351 (NH), 1656 (C=N), 1548,1514 (C=C); ^1^H-NMR (400 MHz, DMSO-*d_6_*): δ = 1.40 (brs, 4H, 2CH_2_), 1.55 (brs, 2H, CH_2_), 3.65 (brs, 4H, 2 NCH_2_-), 4.40–4.45 (m, 2H, CH_2_-NH), 7.14 (t, 1H, *J* = 7.0 Hz, Ar), 7.23–7.29 (m, 4H, Ar), 7.52–7.57 (m, 4H, Ar), 8.00 (s, 1H, CH), 10.72–10.77 (m, 2H, 2NH)) ppm; ^13^C-NMR (100 MHz, DMSO-*d_6_*): δ = 24.9, 25.9, 43.7, 44.1.9, 122.4, 127.0, 127.9, 128.6, 132.2, 134.9, 140.5, 140.9, 164.8, 166.7 ppm. Anal. Calc. for C_22_H_24_BrN_7_ (466.38): C, 56.66; H, 5.19; N, 21.02. Found C, 56.83; H, 5.41; N, 21.27.

*2-(2-benzylidenehydrazinyl)-4-methoxy-6-(piperidin-1-yl)-1,3,5-triazine* (**4d**). the product was obtained as a white solid in yield 81%; mp 221–223 °C; IR (KBr, cm^−1^): 3219 (NH), 1591 (C=N), 1542, 1513 (C=C); ^1^H-NMR (400 MHz, DMSO-*d_6_*): δ = 1.47 (brs, 4H, 2CH_2_), 1.58 (brs, 2H, CH_2_), 3.70 (brs, 4H, 2 NCH_2_-), 3.79 (s, 3H, OCH_3_), 7.31–7.39 (m, 3H, Ar), 7.61 (d, 2H, *J* = 8.25 Hz, Ar), 8.07 (s, 1H, CH), 11.08 (s, 1H, NH) ppm; ^13^C-NMR (100 MHz, DMSO-*d_6_*): δ = 24.8, 25.9, 44.3, 54.1, 127.0, 129.3, 129.8, 135.5, 143.3, 165.3, 171.5 ppm. Anal. Calc. for C_16_H_20_N_6_O (312.37): C, 61.52; H, 6.45; N, 26.90. Found C, 61.71; H, 6.56; N, 27.03.

*2-(2-(4-chlorobenzylidene)hydrazinyl)-4-methoxy-6-(piperidin-1-yl)-1,3,5-triazine* (**4e**). the product was obtained as a white solid in yield 86%; mp 123–125 °C; IR (KBr, cm^−1^): 3219 (NH), 1590 (C=N), 1540, 1462 (C=C);^1^H-NMR (400 MHz, DMSO-*d_6_*): δ = 1.47 (brs, 4H, 2CH_2_), 1.58 (brs, 2H, CH_2_), 3.69 (brs, 4H, 2 NCH_2_-), 3.79 (s, 3H, OCH_3_), 7.43 (d, 2H, *J* = 7.5 Hz, Ar), 7.62 (d, 2H, *J* = 6.5 Hz, Ar), 8.05 (s, 1H, CH), 11.15 (s, 1H, NH) ppm; ^13^C-NMR (100 MHz, DMSO-*d_6_*): δ = 24.7, 25.9, 43.7, 54.1, 128.6, 129.4, 129.6, 130.5, 1341, 134.3, 142.0, 165.8, 171.4 ppm. Anal. Calc. for C_16_H_19_ClN_6_O (346.81): C, 55.41; H, 5.52; N, 24.23. Found C, 55.62; H, 5.68; N, 24.44.

*2-(2-(4-bromobenzylidene)hydrazinyl)-4-methoxy-6-(piperidin-1-yl)-1,3,5-triazine* (**4f**). the product was obtained as a yellow solid in yield 85%; mp 138–140 °C; IR (KBr, cm^−1^): 3217 (NH), 1590 (C=N), 1541, 1462 (C=C); ^1^H-NMR (400 MHz, DMSO-*d_6_*): δ = 1.47 (brs, 4H, 2CH_2_), 1.58 (brs, 2H, CH_2_), 3.69 (brs, 4H, 2CH_2_N), 3.79 (s, 3H, OCH_3_), 7.54–7.59 (m, 4H, Ar), 8.03 (s, 1H, CH), 11.16 (s, 1H, NH) ppm; ^13^C-NMR (100 MHz, DMSO-*d_6_*): δ = 24.7, 25.9, 43.7, 54.1, 122.9, 128.8, 130.7, 132.3, 134.6, 142.1, 165.8, 171.4 ppm. Anal. Calc. for C_16_H_19_BrN_6_O (391.27): C, 49.12; H, 4.89; N, 21.48. Found C, 49.33; H, 4.97; N, 21.67.

*4,4′-(6-(2-(4-fluorobenzylidene)hydrazinyl)-1,3,5-triazine-2,4-diyl)di morpholine* (**4g**). the product was obtained as a white solid in yield 74%; mp 140–142 °C; IR (KBr, cm^−1^): 3253 (NH), 1662 (C=N), 1619, 1510 (C=C); ^1^H-NMR (400 MHz, DMSO-*d_6_*): δ = 3.54 (brs, 4H, 2 OCH_2_-), 3.65 (brs, 4H, 2 NCH_2_-), 4.42–4.48 (m, 2H, CH_2_-NH), 7.16 (t, 1H, *J* = 7.25 Hz, Ar), 7.24–7.31 (m, 4H, Ar), 7.36 (t, 3H, *J* = 7.5 Hz, Ar), 7.59 (d, 2H, *J* = 7.5 Hz, Ar), 8.06 (s, 1H, CH), 10.73 (s, 1H, NH) ppm;^13^C-NMR (100 MHz, DMSO-*d_6_*): δ = 43.2, 43.6, 65.9, 126.4, 126.5, 127.3, 128.1, 128.7, 128.9, 135.1, 140.5, 141.6, 164.2, 164.7, 164.9ppm. Anal. Calc. for: C_21_H_23_N_7_O (389.45): C, 64.76; H, 5.95; N, 25.18. Found C, 64.91; H, 6.03; N, 25.33.

*N-benzyl-4-(2-(4-chlorobenzylidene)hydrazinyl)-6-morpholino-1,3,5-triazin-2-amine* (**4h**). the product was obtained as a yellow solid in yield 77%; mp 260–262 °C; IR (KBr, cm-1): 3272 (NH), 1658 (C=N), 1613, 1513 (C=C); ^1^H-NMR (400 MHz, DMSO-*d_6_*): δ = 3.58 (brs, 4H, 2 OCH_2_-), 3.71 (brs, 4H, 2 NCH_2_-), 4.47–4.51 (m, 2H, CH_2_-NH), 7.26–7.33 (m, 3H, Ar), 7.44 (d, 2H, *J* = 9.0 Hz, Ar), 7.54 (d, 2H, *J* = 8.5 Hz, Ar), 7.86 (d, 2H, *J* = 8.5 Hz, Ar), 8.11 (s, 1H, CH), 10.81 (brs, 1H, NH) ppm; ^13^C-NMR (100 MHz, DMSO-*d_6_*): δ 43.3, 44.1, 66.6, 127.6, 128.0, 128.8, 129.3, 129.6, 130.6, 133.1, 136.6, 140.6, 161.4, 164.2 ppm. Anal. Calc. for: C_21_H_22_ClN_7_O (423.90): C, 59.50; H, 5.23; N, 23.13. Found C, 59.67; H, 5.34; N, 23.31.

*N-benzyl-4-(2-(4-bromobenzylidene)hydrazinyl)-6-morpholino-1,3,5-triazin-2-amine* (**4i**). the product was obtained as a yellow solid in yield 75%; mp 256–258 °C; IR (KBr, cm-1): 3424(NH), 1657(C=N), 1613,1513(C=C);^1^H-NMR (400 MHz, DMSO-*d_6_*): δ = 3.57 (brs, 4H, 2 OCH_2_-), 3.69 (brs, 4H, 2 NCH_2_-), 4.48 (brs, 2H, CH_2_-NH), 7.18–7.32 (m, 5H, Ar), 7.56–7.58 (m, 4H, Ar), 8.08 (s, 1H, CH), 10.81 (s, 1H, NH) ppm; ^13^C-NMR (100 MHz, DMSO- *d_6_*): δ 43.2, 44.1, 66.4, 127.1, 127.5, 128.7, 128.9, 130.7, 131.9, 132.6, 134.2, 137.3, 140.9, 161.3, 164.8 ppm. Anal. Calc. for: C_21_H_22_BrN_7_O (468.35): C, 53.85; H, 4.73; N, 20.93. Found C, 53.61; H, 4.62; N, 21.14.

*4-(4-(2-benzylidenehydrazinyl)-6-methoxy-1,3,5-triazin-2-yl)morpholine* (**4j**). the product was obtained as a white solid in yield 74%; mp 144–146 °C; IR (KBr, cm^−1^): 3353(NH), 1677(C=N), 1583,1537(C=C);^1^H-NMR (400 MHz, DMSO-*d_6_*): δ = 3.60 (brs, 4H, 2 OCH_2_-), 3.70 (brs, 4H, 2 NCH_2_-), 3.80 (s, 3H, OCH_3_), 7.32–7.39 (m, 3H, Ar), 7.62 (d, 2H, *J* = 6.5 Hz, Ar), 8.08 (s, 1H, CH), 11.17 (s, 1H, NH) ppm; ^13^C-NMR (100 MHz, DMSO- *d_6_*): δ 43.4, 53.7, 65.9, 126.6, 128.7, 129.3, 134.7, 143.2, 165.3, 165.7 ppm. Anal. Calc. for: C_15_H_18_N_6_O_2_ (314.34): C, 57.31; H, 5.77; N, 26.74. Found C, 57.55; H, 5.89; N, 26.93.

*4-(4-(2-(4-chlorobenzylidene)hydrazinyl)-6-methoxy-1,3,5-triazin-2-yl)morpholine* (**4k**). the product was obtained as a white solid in yield 77%; mp 240–242 °C; IR (KBr, cm-1): 3220(NH), 1588(C=N), 1542,1460(C=C); ^1^H-NMR (400 MHz, DMSO-*d_6_*): δ = 3.60 (brs, 4H, 2 OCH_2_-), 3.69 (brs, 4H, 2 NCH_2_-), 3.80 (s, 3H, OCH_3_), 7.44 (dd, 2H, *J* = 6.5, 2.5 Hz, Ar), 7.63 (dd, 2H, *J* = 6.5, 2.5 Hz, Ar), 8.06 (s, 1H, CH), 11.24 (s, 1H, NH) ppm; ^13^C-NMR (100 MHz, DMSO- *d_6_*): δ 43.4, 54.2, 66.4, 128.7, 129.4, 130.6, 134.2, 142.4, 161.6, 165.8 ppm. Anal. Calc. for: C_15_H_17_ClN_6_O_2_ (348.79): C, 51.65; H, 4.91; N, 24.09. Found C, 51.82; H, 4.83; N, 24.31.

*4-(4-(2-(4-bromobenzylidene)hydrazinyl)-6-methoxy-1,3,5-triazin-2-yl)morpholine* (**4l**). the product was obtained as a white solid in yield 76%; mp 234–236 °C; IR (KBr, cm-1): 3228(NH), 1584(C=N), 1538,1462(C=C); ^1^H-NMR (400 MHz, DMSO-*d_6_*): δ = 3.64 (d, 4H, *J* = 4.4 Hz, 2 OCH_2_-), 3.73 (brs, 4H, 2 NCH_2_-), 3.83 (s, 3H, OCH_3_), 7.59 (s, 4H, Ar), 8.07 (s, 1H, CH), 11.26 (s, 1H, NH) ppm; ^13^C-NMR (100 MHz, DMSO-*d_6_*): δ 43.4, 53.7, 65.9, 122.4, 128.3, 131.7, 134.0, 141.9, 164.8, 166.4 ppm. Anal. Calc. for: C_15_H_17_BrN_6_O_2_ (393.24): C, 45.81; H, 4.36; N, 21.37. Found C, 45.99; H, 4.51; N, 21.59.

*4-(2-benzylidenehydrazinyl)-6-morpholino-N-phenyl-1,3,5-triazin-2-amine* (**4m**). the product was obtained as a white solid in yield 84%; mp 244–246 °C; IR (KBr, cm^−1^): 3259(NH), 1608(C=N), 1551,1503(C=C); ^1^H-NMR (400 MHz, DMSO-*d_6_*): δ = 3.65 (d, 4H, *J* = 4.4 Hz, 2 OCH_2_-), 3.75 (brs, 4H, 2 NCH_2_-), 6.95 (t, 1H, *J* = 7.6 Hz, Ar), 7.27 (t, 2H, *J* = 8.0 Hz, Ar), 7.36–7.45 (m, 3H, Ar), 7.67 (d, 2H, *J* = 8.5 Hz, Ar), 7.83 (brs, 2H, Ar), 8.14 (s, 1H, CH), 9.30 (brs, 1H, NH), 10.92 (brs, 1H, NH) ppm; ^13^C-NMR (100 MHz, DMSO-*d_6_*): δ 43.2, 66.0, 119.7, 121.6, 126.5, 128.4, 128.7, 129.1, 135.1, 140.3, 142.2, 164.2, 164.8 ppm. Anal. Calc. for: C_20_H_21_N_7_O (375.43): C, 63.98; H, 5.64; N, 26.12. Found C, 64.08; H, 5.77; N, 26.36.

*4-(2-(4-chlorobenzylidene)hydrazinyl)-6-morpholino-N-phenyl-1,3,5-triazin-2-amine* (**4n**). the product was obtained as a white solid in yield 84%; mp 258–260 °C; IR (KBr, cm-1): 3253(NH), 1586(C=N), 1544, 1510 (C=C); ^1^H-NMR (400 MHz, DMSO-*d_6_*): δ = 3.65 (d, 4H, *J* = 4.4 Hz, 2 OCH2-), 3.75 (brs, 4H, 2 NCH_2_-), 6.95 (t, 1H, *J* = 7.6 Hz, Ar), 7.28 (t, 2H, *J* = 7.8 Hz, Ar), 7.50 (d, 2H, *J* = 8.0 Hz, Ar), 7.68 (d, 2H, *J* = 8.8 Hz, Ar), 7.82 (brs., 2H, Ar), 8.12 (s, 1H, CH), 9.33 (brs., 1H, NH), 10.99 (brs., 1H, NH) ppm; ^13^C-NMR (100 MHz, DMSO- *d_6_*): δ 43.4, 66.0, 119.8, 121.5, 127.8, 128.1, 128.9, 133.4, 133.9, 140.3, 141.0, 164.1, 164.2, 164.8 ppm. Anal. Calc. for: C_20_H_20_ClN_7_O (409.87): C, 58.61; H, 4.92; N, 23.92. Found C, 58.79; H, 5.04; N, 24.12.

*4-(2-(4-bromobenzylidene)hydrazinyl)-6-morpholino-N-phenyl-1,3,5-triazin-2-amine* (**4o**). the product was obtained as a white solid in yield 85%; mp 259–261 °C; IR (KBr, cm^−1^): 3249 (NH), 1586(C=N), 1547, 1510 (C=C); ^1^H-NMR (400 MHz, DMSO-*d_6_*): δ = 3.65 (d, 4H, *J* = 4.4 Hz, 2 OCH2-), 3.75 (brs, 4H, 2 NCH_2_-), 6.94 (t, 1H, *J* = 7.4 Hz, Ar), 7.27 (t, 2H, *J* = 8.2 Hz, Ar), 7.62 (brs, 4H, Ar), 7.82 (brs, 2H, Ar), 8.11 (s, 1H, CH), 9.34 (brs, 1H, NH), 10.99 (brs., 1H, NH) ppm; ^13^C-NMR (100 MHz, DMSO-d_6_): δ = 43.4, 66.0, 119.8, 121.6, 122.1, 128.3, 131.7, 134.3, 140.3, 141.1, 164.2, 164.8 ppm. Anal. Calc. for C_20_H_20_BrN_7_O (454.32): C, 52.87; H, 4.44; N, 21.58. Found C, 53.03; H, 4.54; N, 21.79.

*4-(2-benzylidenehydrazinyl)-6-methoxy-N-phenyl-1,3,5-triazin-2-amine* (**4p**). the product was obtained as a white solid in yield 83%; mp 148–150 °C; IR (KBr, cm^−1^): 3254(NH), 1590(C=N), 1506, 1423(C=C); ^1^H-NMR (400 MHz, DMSO-*d_6_*): δ = 3.68 (s, 3H, OCH_3_), 6.81 (d, 3H, *J* = 9.5 Hz, Ar), 7.30–7.40 (m, 3H, Ar), 7.62 (d, 2H, *J* = 7.5 Hz, Ar), 7.70 (brs, 2H, Ar), 8.11 (s, 1H, CH), 9.05 (brs., 1H, NH), 10.73 (brs, 1H, NH) ppm; ^13^C-NMR (100 MHz, DMSO-*d_6_*): δ = 55.1, 113.5, 120.9, 126.3, 128.6,132.9, 133.7, 135.2, 141.7, 154.1, 164.0, 164.2ppm. Anal. Calc. for C_17_H_16_N_6_O (320.35): C, 63.74; H, 5.03; N, 26.23. Found C, 63.93; H, 5.13; N, 26.47.

*4-(2-(4-chlorobenzylidene)hydrazinyl)-6-methoxy-N-phenyl-1,3,5-triazin-2-amine* (**4q**). the product was obtained as a white solid in yield 81%; mp 178–180 °C; IR (KBr, cm-1): 3346(NH), 1649(C=N), 1515,1417(C=C); ^1^H-NMR (400 MHz, DMSO-*d_6_*): δ = 3.68 (s, 3H, OCH_3_), 6.81 (d, 3H, *J* = 9.0 Hz, Ar), 7.46 (d, 2H, *J* = 8.5 Hz, Ar), 7.62–7.69 (m, 4H, Ar), 8.09 (s, 1H, CH), 9.07 (brs, 1H, NH), 10.80 (brs., 1H, NH) ppm; ^13^C-NMR (100 MHz, DMSO- *d_6_*): δ 55.1, 113.5, 120.9, 127.8, 128.8, 133.2, 133.7, 134.2, 140.3, 154.1, 164.0, 164.2 ppm. Anal. Calc. for: C_17_H_15_ClN_6_O (354.79): C, 57.55; H, 4.26; N, 23.69. Found C, 57.79; H, 4.37; N, 23.91.

*3.1.18. 4-(2-(4-bromobenzylidene)hydrazinyl)-6-methoxy-N-phenyl-1,3,5-triazin-2-amine* (**4r**). the product was obtained as a white solid in yield 77%; mp 173–175 °C; IR (KBr, cm-1): 3333(NH), 1655(C=N), 1556,1515(C=C);^1^H-NMR (400 MHz, DMSO-*d_6_*): δ = 3.71 (s, 3H, OCH_3_), 6.84 (d, 3H, *J* = 6.5 Hz, Ar), 6.85–7.72 (m, 6H, Ar), 8.11 (s, 1H, CH), 9.10 (brs., 1H, NH), 10.83 (brs, 1H, NH) ppm; ^13^C-NMR (100 MHz, DMSO- *d_6_*): δ 55.1, 113.5, 120.9, 121.9, 128.1, 131.7, 133.7, 134.5, 140.4, 154.1, 164.0, 164.2 ppm. Anal. Calc. for: C_17_H_15_BrN_6_O (399.24): C, 51.14; H, 3.79; N, 21.05. Found C, 51.31; H, 3.92; N, 21.28.

### 3.2. Biology

#### 3.2.1. In Vitro Anti-Proliferative Assay

The synthesized compounds **4a**–**r** were evaluated for anti-proliferative using the MTT viability assay of MCF-7 and HCT-116 cell lines and to calculate the relative IC_50_ values for each compound as illustrated in the (Supporting information, Method S1).

#### 3.2.2. Annexin V/PI Apoptotic Assay:

Apoptotic cell death was detected by flow cytometry using Annexin V and propidium iodide (PI). MCF-7 cells were seeded in 6 well plated at density of 1 × 10^5^ cells/mL and treated with vehicle (0.1% (v/v) DMSO) and compound **4b** (6 µM) for 24 h. Cells were then harvested and prepared for flow cytometric analysis as illustrated in the (Supporting information, Method S2).

#### 3.2.3. Molecular Docking Study

The 3ERT X-ray structure of hERα co-crystallized with 4-hydroxytamoxifen (4-OHT) [44] was downloaded from the protein data bank (PDB) website. The crystal structure was prepared using QuickPrep (minimized to a gradient of 0.001 kcal/mol/Å), Protonate 3D, Residue pKa and Partial Charges protocols in MOE 2019 [45] with the MMFF94x force field. All compounds were drawn in ChemBioDraw Ultra 13.0.2.3021 (PerkinElmer), converted to sd files within Pipeline Pilot [46] and read into a MOE mdb file. For each compound, MMFF94x partial charges were calculated and each was minimized to a gradient of 0.001 kcal/mol/Å. Default parameters were used for docking except that 300 poses were sampled for each compound and the top 50 docked poses were retained for subsequent analysis.

## 4. Conclusions

In summary, the synthesized compounds in this study showed varied activity against two cell lines MCF-7 and HCT-116. Generally, the nature of the substituents on the triazine ring significantly influenced the biological activity. Compounds with piperidine and benzylamine moieties on the triazine core **4a-c** showed the most potent anti-proliferative activity with IC_50_ values in range of 3.29–11.35 µM and 3.64 -12.45 µM for MCF-7 and HCT-116 cells, respectively compared with tamoxifen as a reference compound (IC_50_ values of 5.12 and 26.41 µM in MCF-7 and HCT-116 cells, respectively) and their analogs with substituted morpholine and benzylamine **4g**–**i** (IC_50_ values in range of 16.44–24.46 µM and 8.19 -14.01 µM in MCF-7 and HCT-116 cells, respectively). Furthermore, compounds **4m**–**o** (morpholine and phenylamine) exhibited better activity compared to their analogous **4p**–**r** (methoxy and phenylamine). In addition, the substituent on the benzylidene ring showed great impact on the anti-proliferative activity, where the chloro derivatives showed better anti-cancer activities compared to other unsubstituted and bromo substituent analogs. The obtained results are consistent with the previously reported results, where the presence of piperidine on the *s*-triazine ring and the presence of an electronegative atom on the benzylidene increased the compounds activity against the cancer cells [32,33,34,35].

The results demonstrate that derivative **4b** can induce apoptosis in MCF-7 cells. Indeed, the results obtained from the molecular docking agreed with the anti-proliferative activity assay, where the combination of the piperidino and benzylamino on the *s*-triazine core is the optimal configuration.

Plans for future optimization of the triazine compound series ER binding affinity include introducing hydrogen bonding acceptor moieties *ortho* and/or *meta* on the halogenated benzylidene ring and hydrogen bond donating moieties *para* on the benzyl ring so as to more fully mimic the 4-OHT binding interactions.

## Figures and Tables

**Scheme 1:**
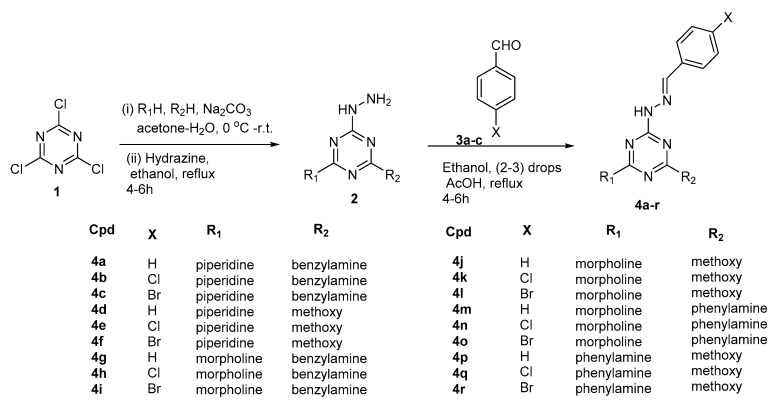
Synthetic route for preparation of *s*-triazine Schiff base derivatives.

**Table 1:** Anti-proliferative effects of **4a**–**q** series compounds in MCF-7 and HCT-116 cells.

Compound no.	IC_50_ (μM) ^a^ MCF-7	IC_50_ (μM)^a^ HCT-116
4a	11.35 ± 1.81	12.45 ± 1.16
4b	3.29 ± 0.83	3.64 ± 1.58
4c	4.63 ± 0.40	5.60 ± 1.97
4d	28.62 ± 2.80	>50
4e	33.90 ± 0.94	>50
4f	18.49 ± 1.03	>50
4g	24.46 ± 1.83	14.01 ± 1.61
4h	16.44 ± 1.13	8.19 ± 0.22
4i	22.20 ± 2.59	12.22 ± 0.34
4j	18.59 ± 1.62	24.10 ± 3.94
4k	14.08 ± 0.06	42.34 ± 4.74
4l	26.91 ± 1.20	37.29 ± 0.34
4m	7.93 ± 0.77	5.10 ± 1.01
4n	6.10 ± 0.42	4.54 ± 0.41
4o	6.58 ± 0.65	10.71± 2.91
4p	3.98 ± 0.22	8.45 ± 0.24
4q	9.37 ± 1.74	6.26 ± 0.49
4r	10.44 ± 0.43	7.57 ± 1.34
Tamoxifen ^b^	5.12 ± 0.36	26.41 ± 4.11

^a^IC_50_ values are half maximal inhibitory concentrations required to block the growth stimulation of cells. Values represent the mean for three experiments performed in triplicate; ^b^The IC_50_ value obtained for CA-4 (5.12 μM for MCF-7 and 26.41 μM for HCT-116) are in good agreement with reported values [39,40].

## Supplementary Materials

The following are available online: https://www.mdpi.com/1420-3049/25/18/4065/s1, Figures S1–S18: represented the spectral data (^1^H and ^13^C-NMR spectra) for all prepared compounds.
