# Supplementary material for: Synthesis, Anti-proliferative Activity, and Molecular Docking Study of New Series of 1,3-5-Triazine Schiff Base Derivatives
Source: Molecules. 2020 Sep 5;25(18):4065. doi: 10.3390/molecules25184065 (PMC7571070; doi:10.3390/molecules25184065)

Supporting Information

Synthesis, Anti-proliferative Activity, and Molecular Docking Study of New Series of 1,3-5-Triazine Schiff Base Derivatives

Hessa H. Al Rasheed,* **Azizah M. Malebari, Kholood A. Dahlous, Darren Fayne, Ayman El-Faham***

Table of Content

Method S1 *In vitro* **anti-proliferative assay**
Method S2 **Annexin V/PI Apoptotic Assay**
Figure S1 ^{1}H-NMR and ^{13}C-NMR for compound **4a**
Figure S2 ^{1}H-NMR and ^{13}C-NMR for compound **4b**
Figure S3 ^{1}H-NMR and ^{13}C-NMR for compound **4c**
Figure S4 ^{1}H-NMR and ^{13}C-NMR for compound **4d**
Figure S5 ^{1}H-NMR and ^{13}C-NMR for compound **4e**
Figure S6 ^{1}H-NMR and ^{13}C-NMR for compound **4f**
Figure S7 ^{1}H-NMR and ^{13}C-NMR for compound **4g**
Figure S8 ^{1}H-NMR and ^{13}C-NMR for compound **4h**
Figure S9 ^{1}H-NMR and ^{13}C-NMR for compound **4i**
Figure S10 ^{1}H-NMR and ^{13}C-NMR for compound **4j**
Figure S11 ^{1}H-NMR and ^{13}C-NMR for compound **4k**
Figure S12 ^{1}H-NMR and ^{13}C-NMR for compound **4l**
Figure S13 ^{1}H-NMR and ^{13}C-NMR for compound **4m**
Figure S14 ^{1}H-NMR and ^{13}C-NMR for compound **4n**
Figure S15 ^{1}H-NMR and ^{13}C-NMR for compound **4o**
Figure S16 ^{1}H-NMR and ^{13}C-NMR for compound **4p**
Figure S17 ^{1}H-NMR and ^{13}C-NMR for compound **4q**
Figure S18 ^{1}H-NMR and ^{13}C-NMR for compound **4r**

Method S1: In vitro anti-proliferative assay

The synthesized compounds **4a-r** were evaluated for anti-proliferative using the MTT viability assay of MCF-7 and HCT-116 cell lines and to calculate the relative IC_{50} values for each compound). Cells were seeded in triplicate in 96-well plates at a density of 10 x 10^3 cells /mL in a total volume of 200 μL per well. 1% ethanol (v/v) was used as a vehicle control. After 24 h, they were treated with 2 μL test compound which had been pre-prepared as stock solutions to furnish the concentration range of study, 0.1 μM to 100 μM, and re-incubated for a further 72 h. The culture medium was then removed and the cells washed with 100 μL phosphate buffered saline (PBS) and 100 μL MTT added, to reach a final concentration of 5 mg/mL MTT added. Cells were incubated for 3 h in darkness at 37 °C. At this point solubilisation was begun through the addition of 200 μL DMSO and the cells maintained at room temperature in darkness for 20 min to ensure thorough color diffusion before reading the absorbance the optical density was detected with a microplate reader at 570 nm. Results were expressed as percentage viability relative to vehicle control (100%). Dose response curves were plotted and IC_{50} values (concentration of drug resulting in 50% reduction in cell survival) were obtained using the commercial software package Prism (GraphPad Software, Inc., La Jolla, CA, USA). All the experiments were repeated in at least three independent experiments.

Method S2: Annexin V/PI Apoptotic Assay

Apoptotic cell death was detected by flow cytometry using Annexin V and propidium iodide (PI). MCF-7 Cells were seeded in 6 well plated at density of 1 × 10^5 cells/mL and treated with vehicle (0.1 % (v/v) DMSO) and compound **4b** (6 μM) for 24 hr. Cells were then harvested and prepared for flow cytometric analysis. Cells were washed in 1X binding buffer (20X binding buffer: 0.1M HEPES, pH 7.4; 1.4 M NaCl; 25 mM $CaCl_2$ diluted in dH2O) and incubated in the dark for 30 minutes on ice in Annexin V-containing binding buffer [1:100]. Cells were then washed once in binding buffer and then re-suspended in PI-containing binding buffer [1:1000]. Samples were analysed immediately using the BD accuri flow cytometer and prism software for analysis the data. Four populations are produced during the assay Annexin V and PI negative (Q4, healthy cells), Annexin V positive and PI negative (Q3, early apoptosis), Annexin V and PI positive (Q2, late apoptosis) and Annexin V negative and PI positive (Q1, necrosis).

Figure S1: ^{1}H-NMR and ^{13}C-NMR for compound **4a**

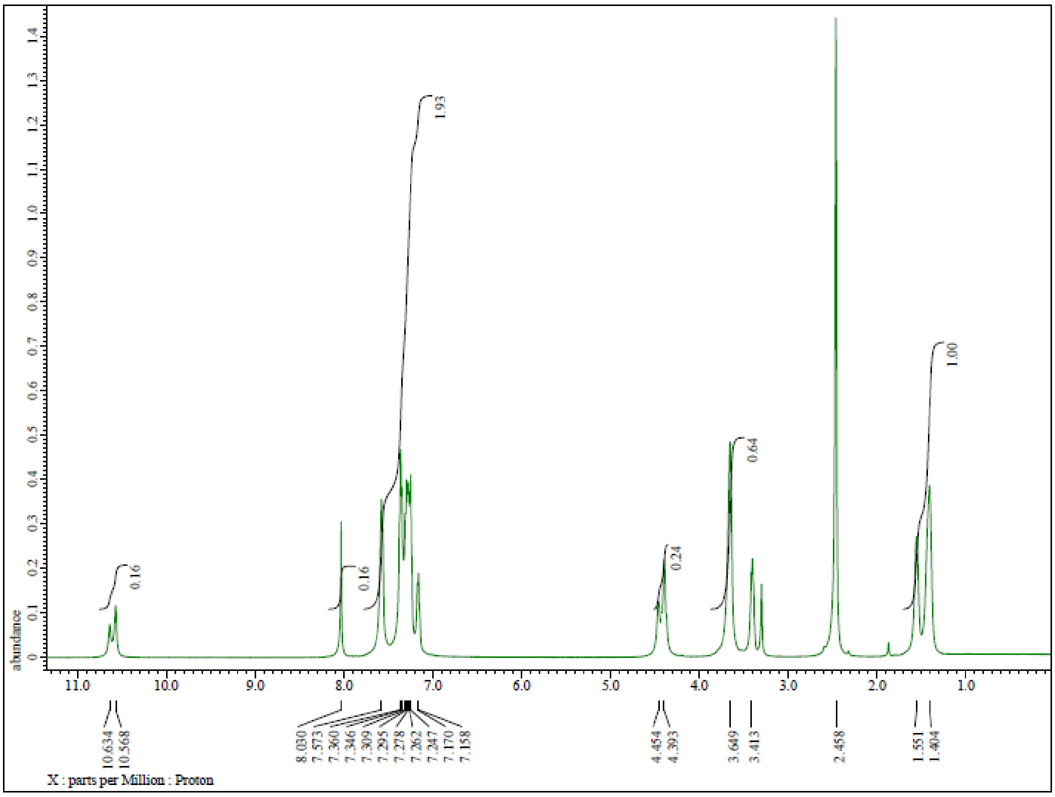

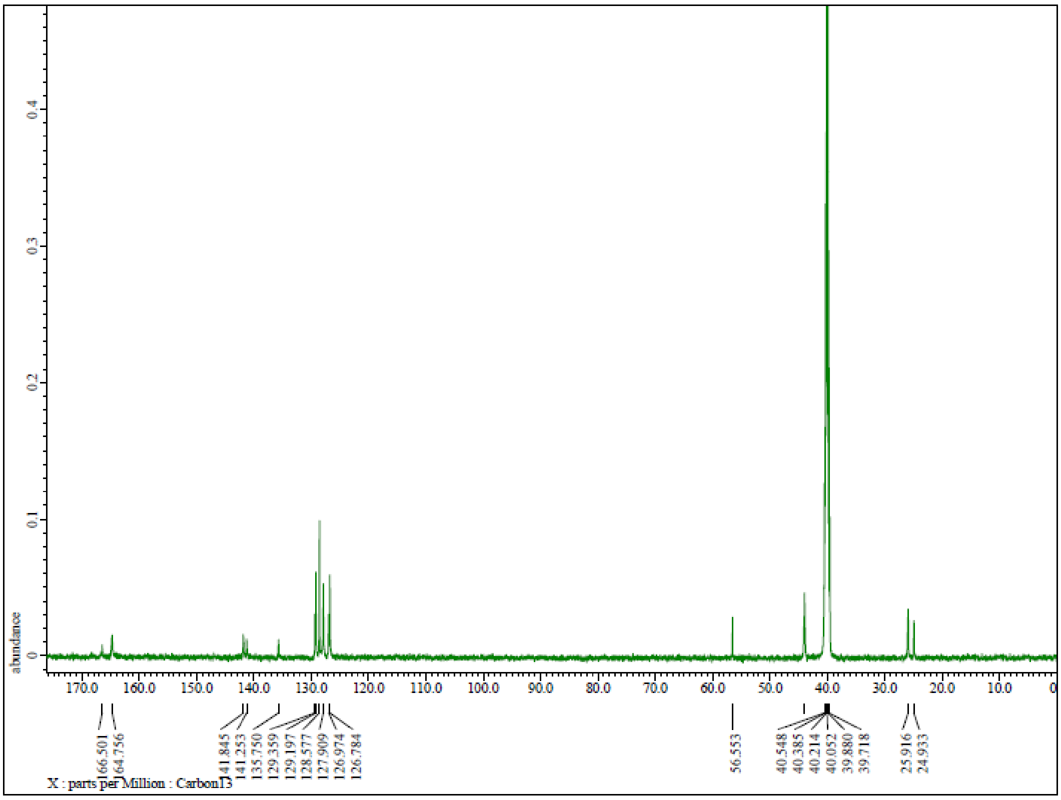

Figure S2: ^{1}H-NMR and ^{13}C-NMR for compound **4b**

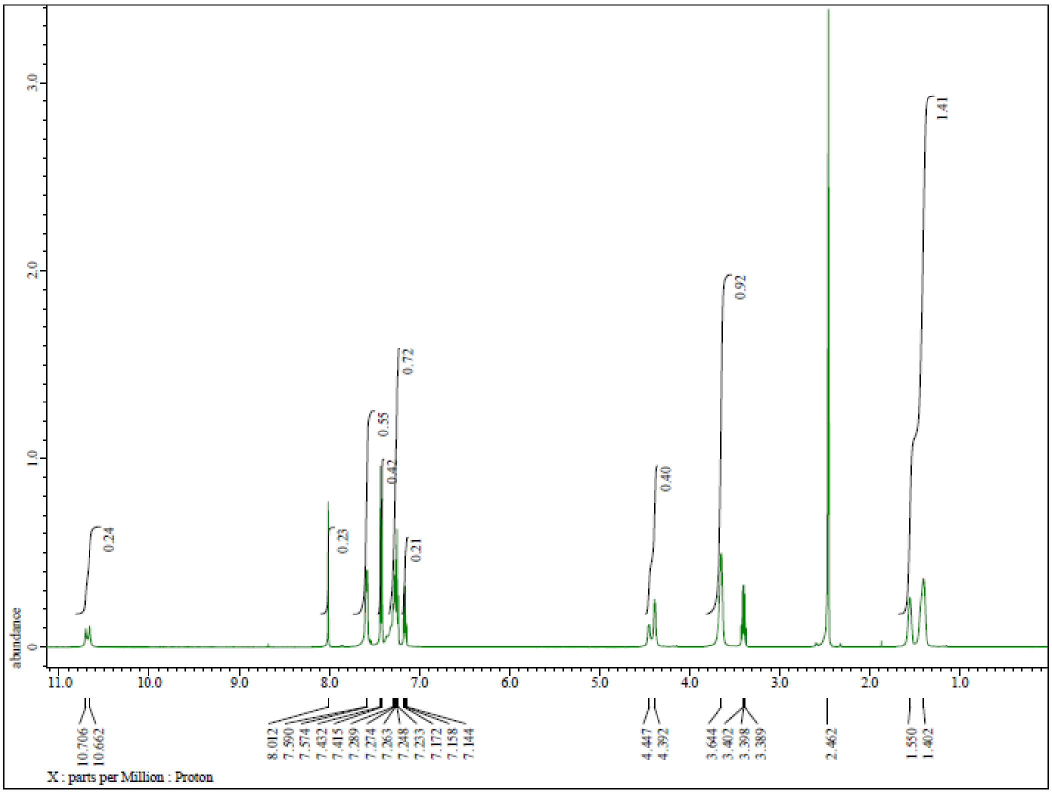

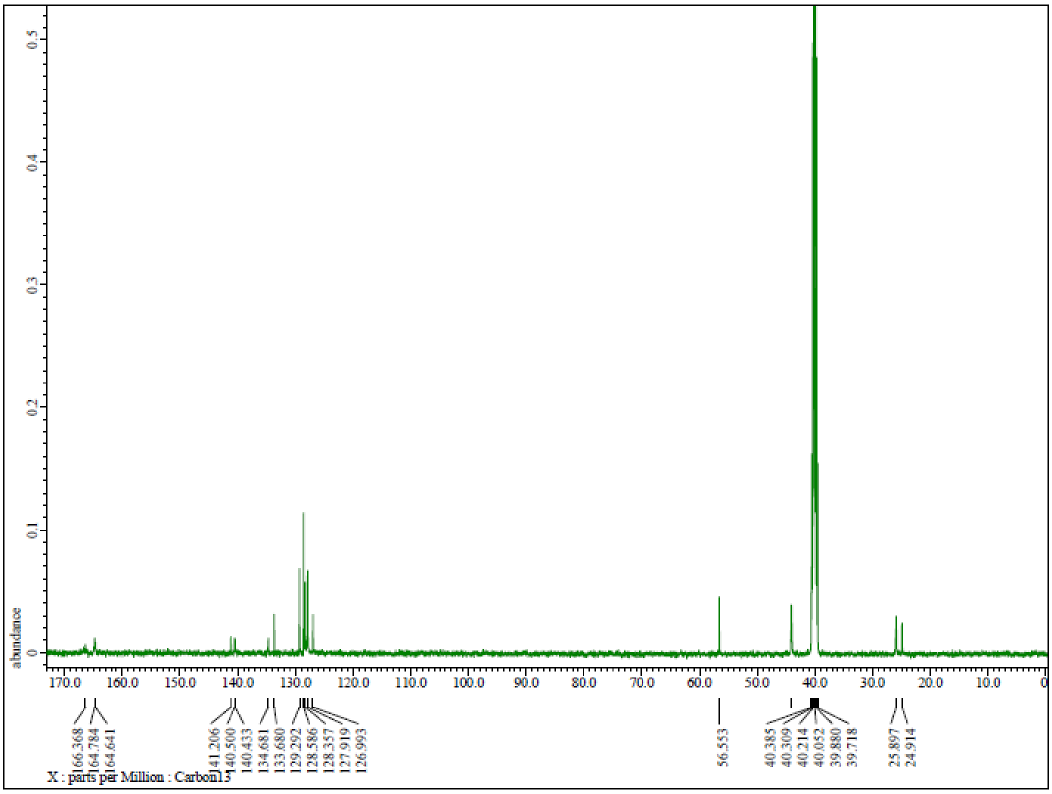

Figure S3: ^{1}H-NMR and ^{13}C-NMR for compound **4c**

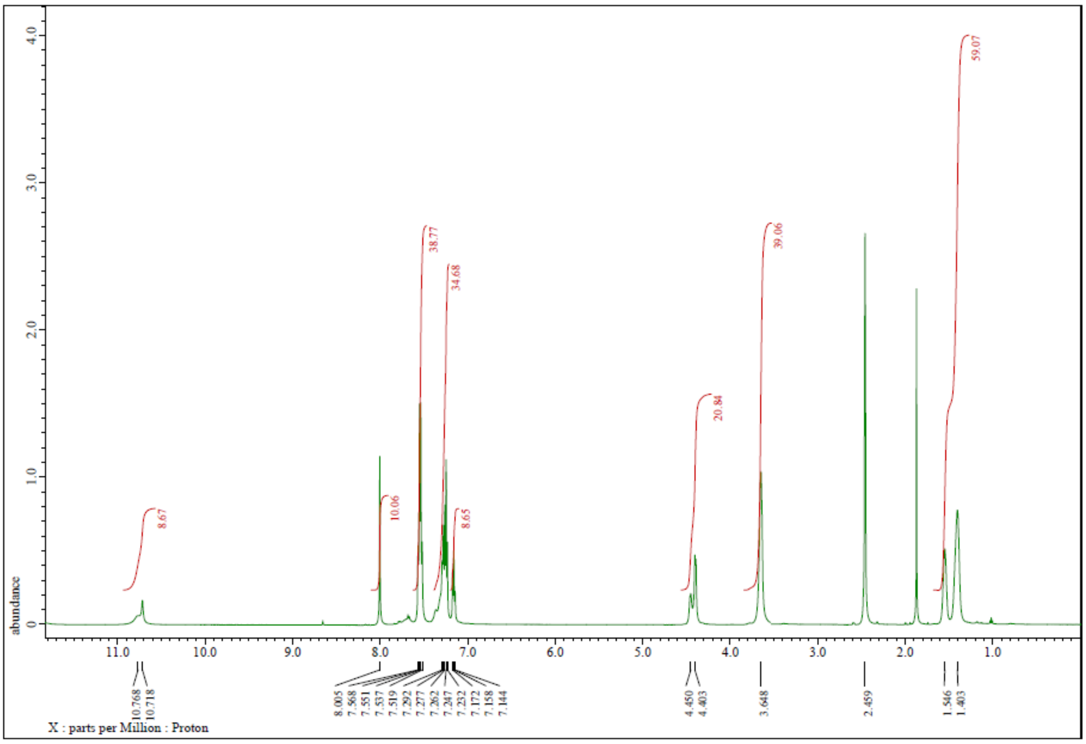

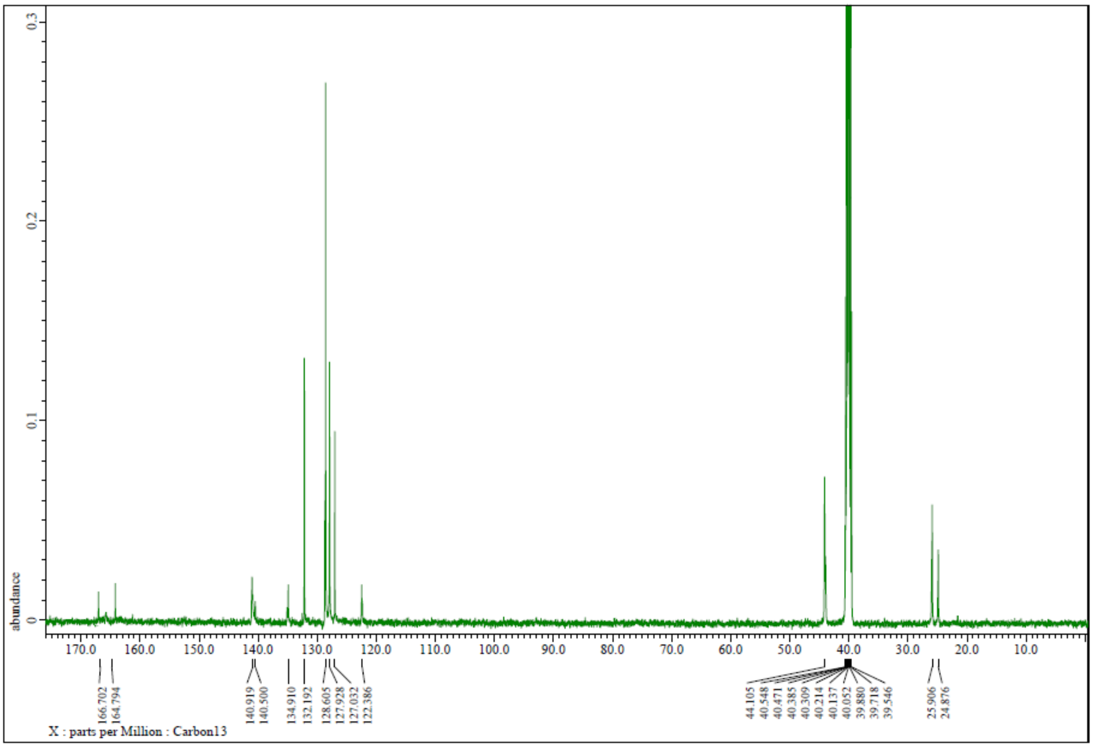

Figure S4: ^{1}H-NMR and ^{13}C-NMR for compound **4d**

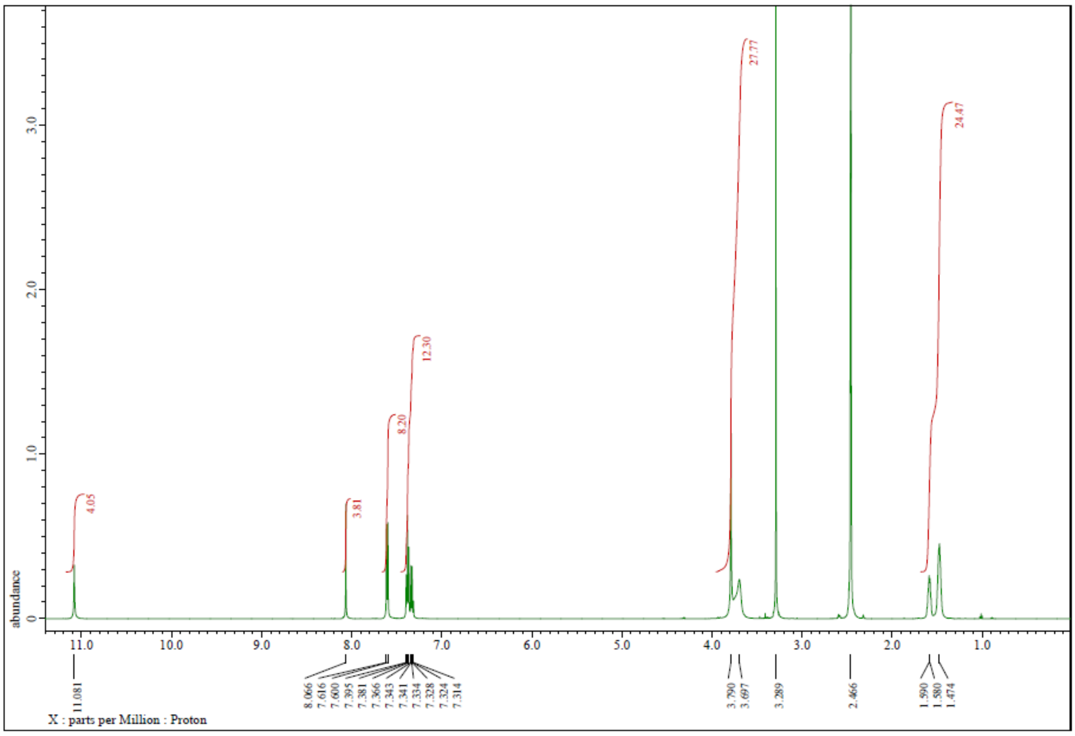

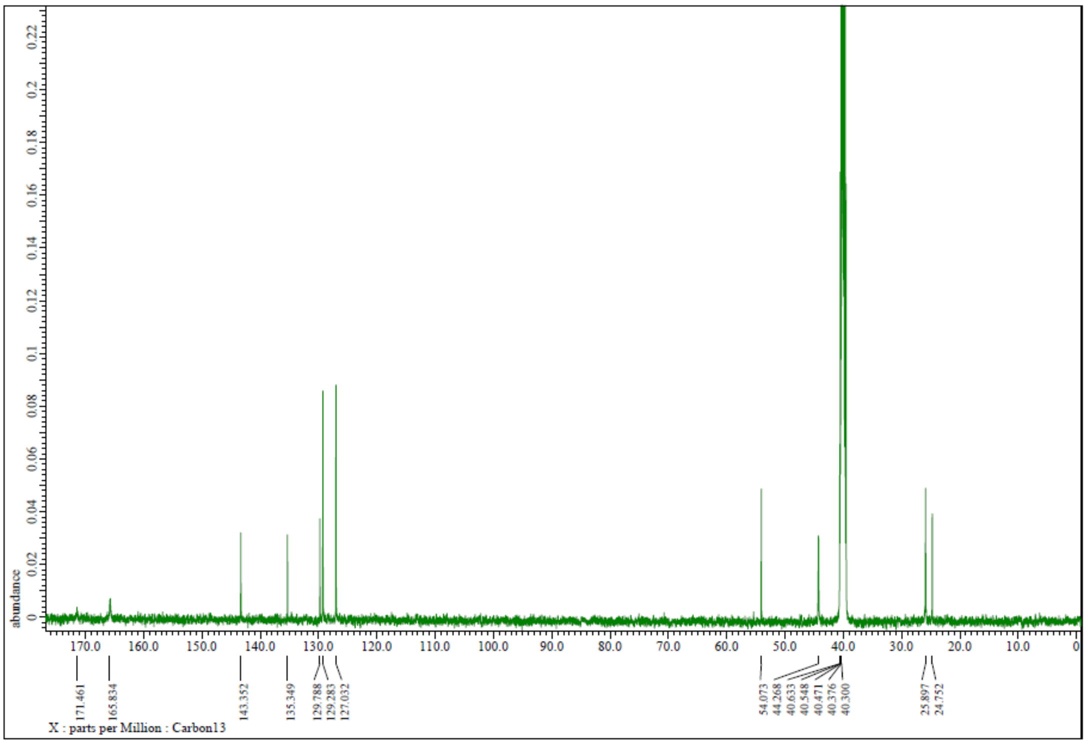

Figure S5: ^{1}H-NMR and ^{13}C-NMR for compound **4e**

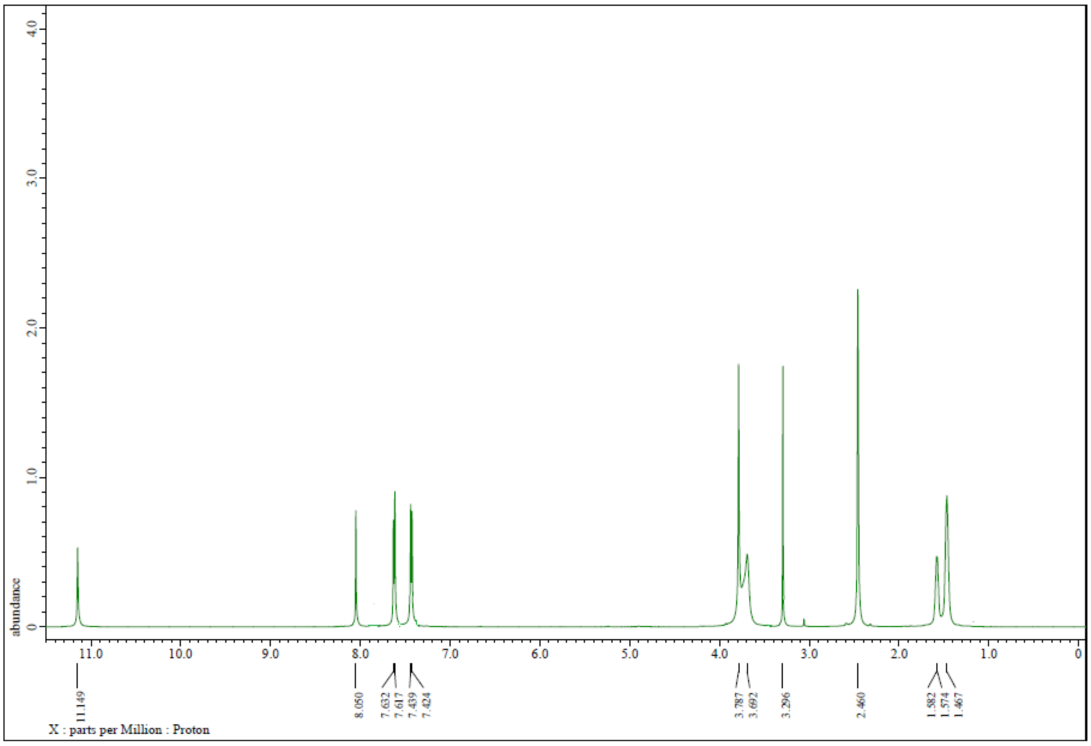

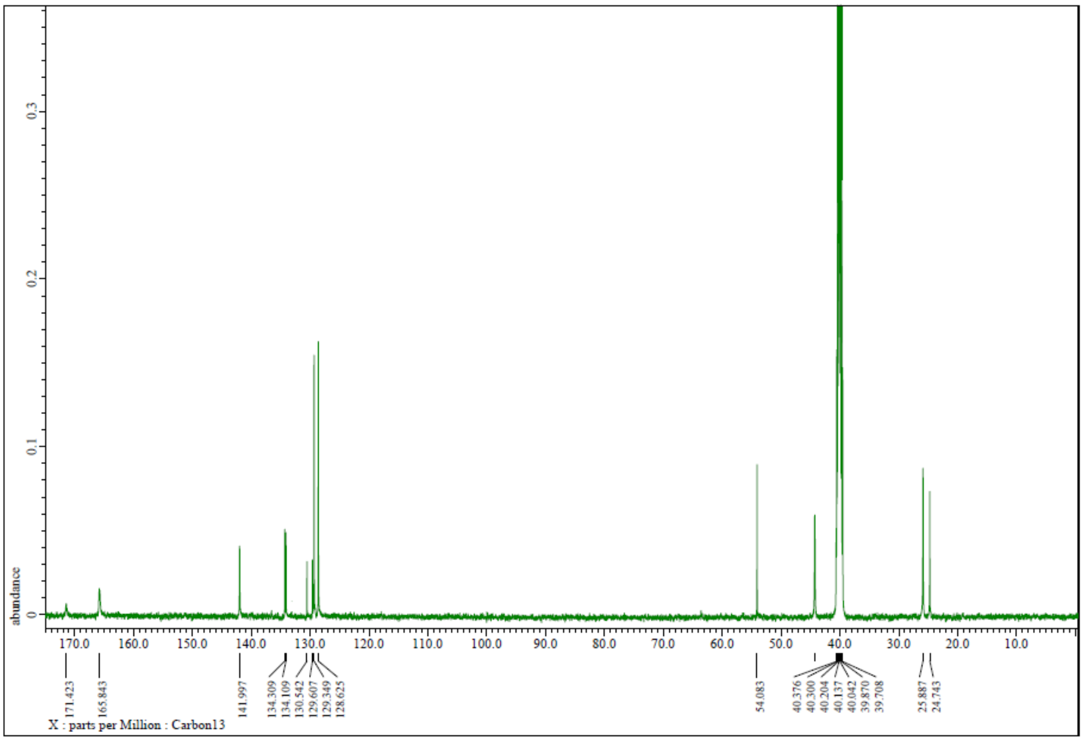

Figure S6: ^{1}H-NMR and ^{13}C-NMR for compound **4f**

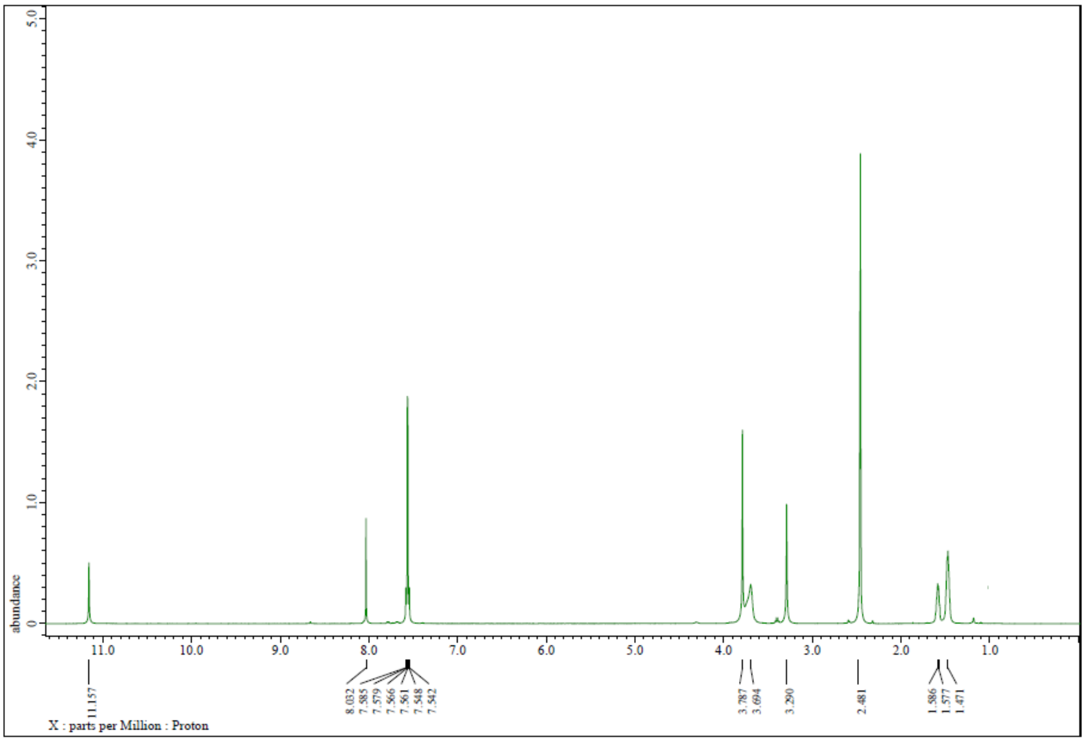

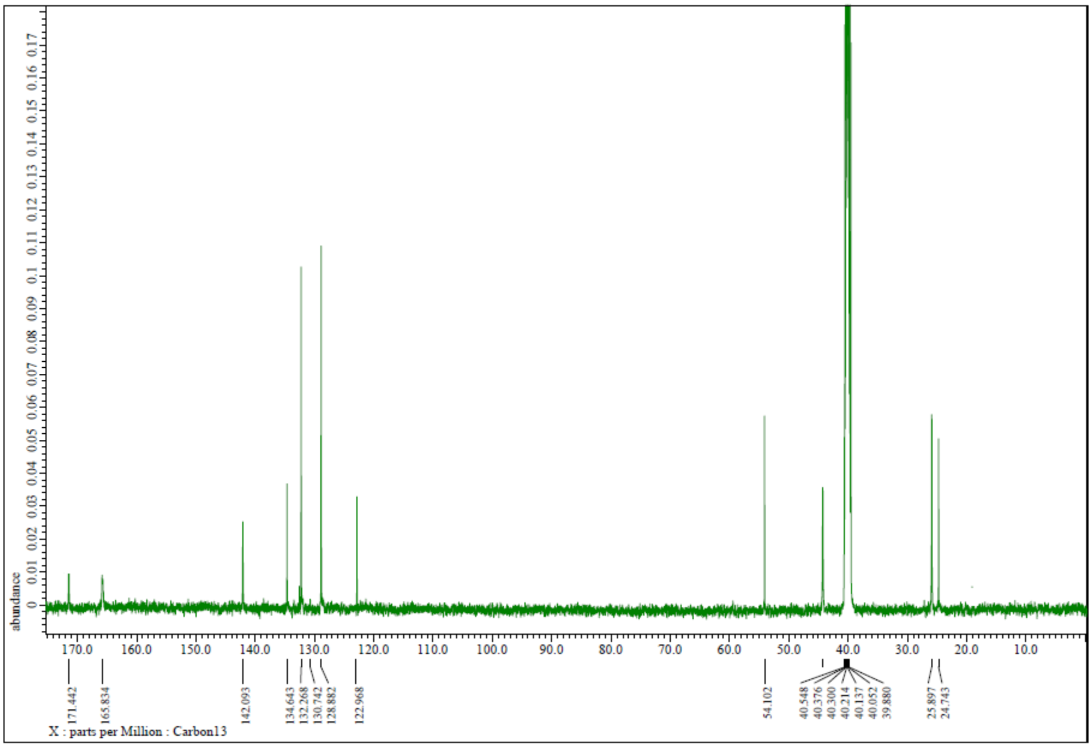

Figure S7: ^{1}H-NMR and ^{13}C-NMR for compound **4g**

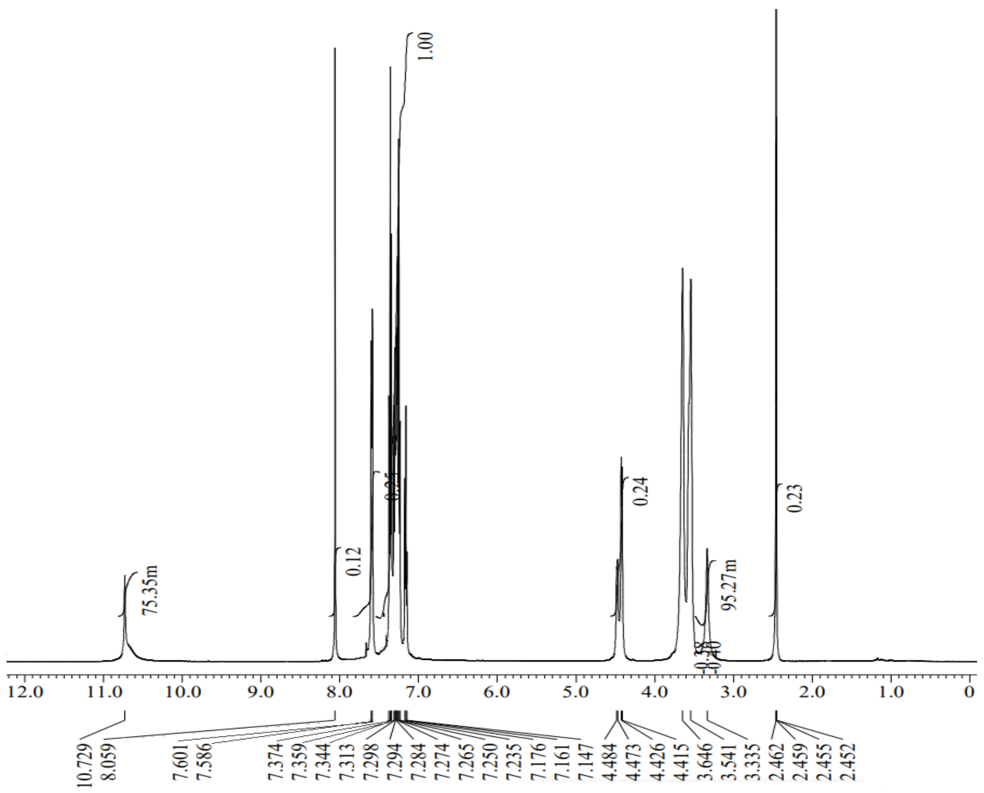

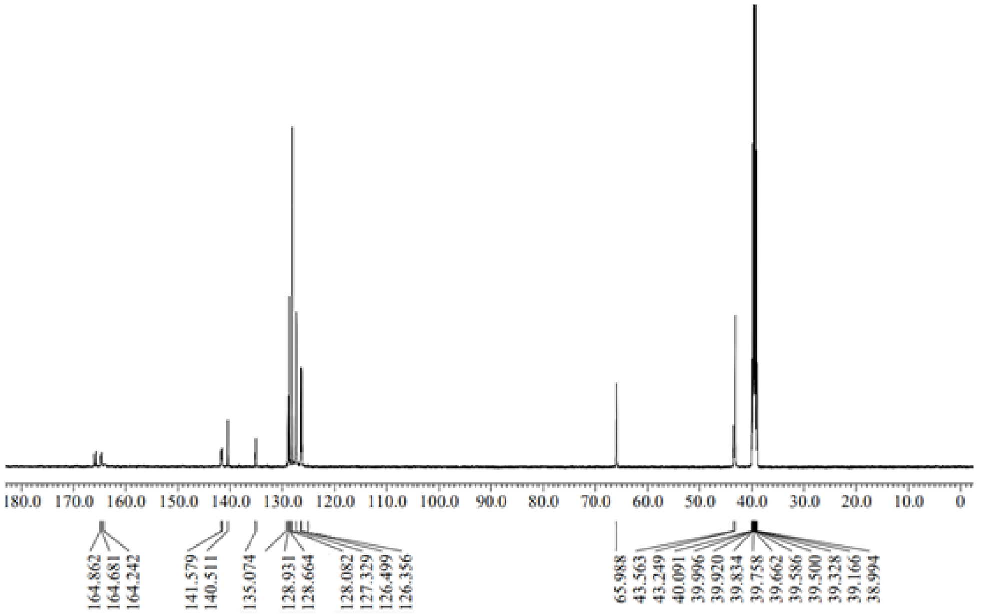

Figure S8: ^{1}H-NMR and ^{13}C-NMR for compound **4h**

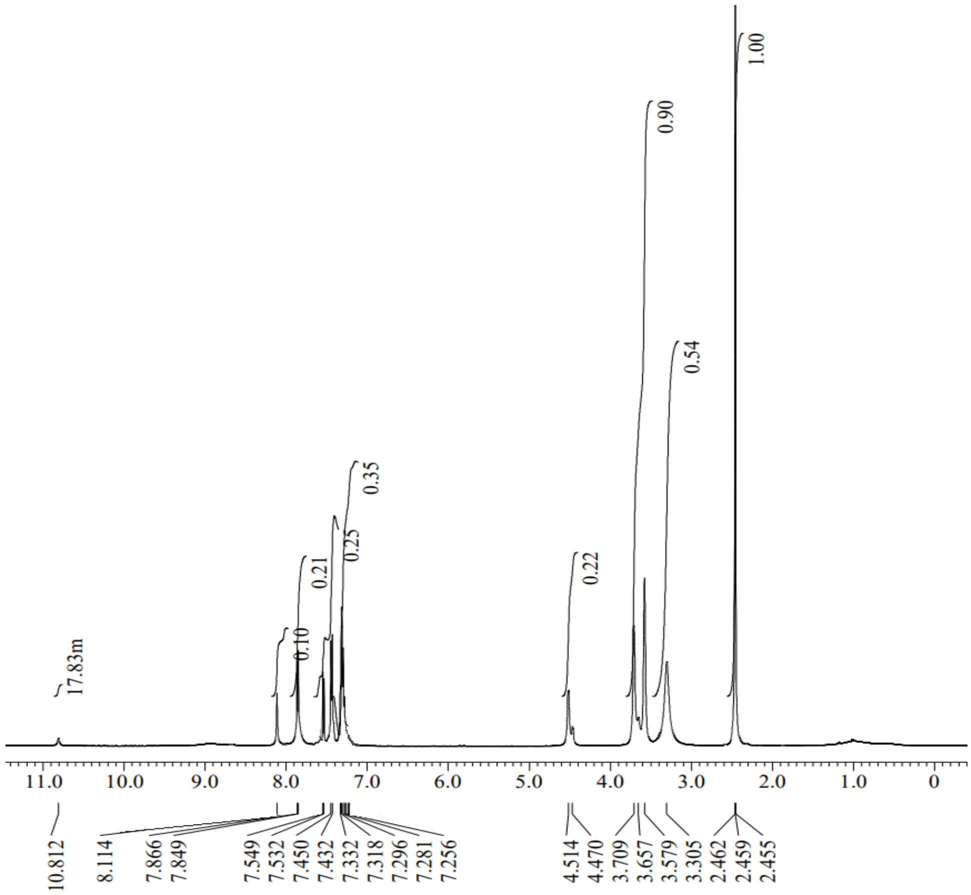

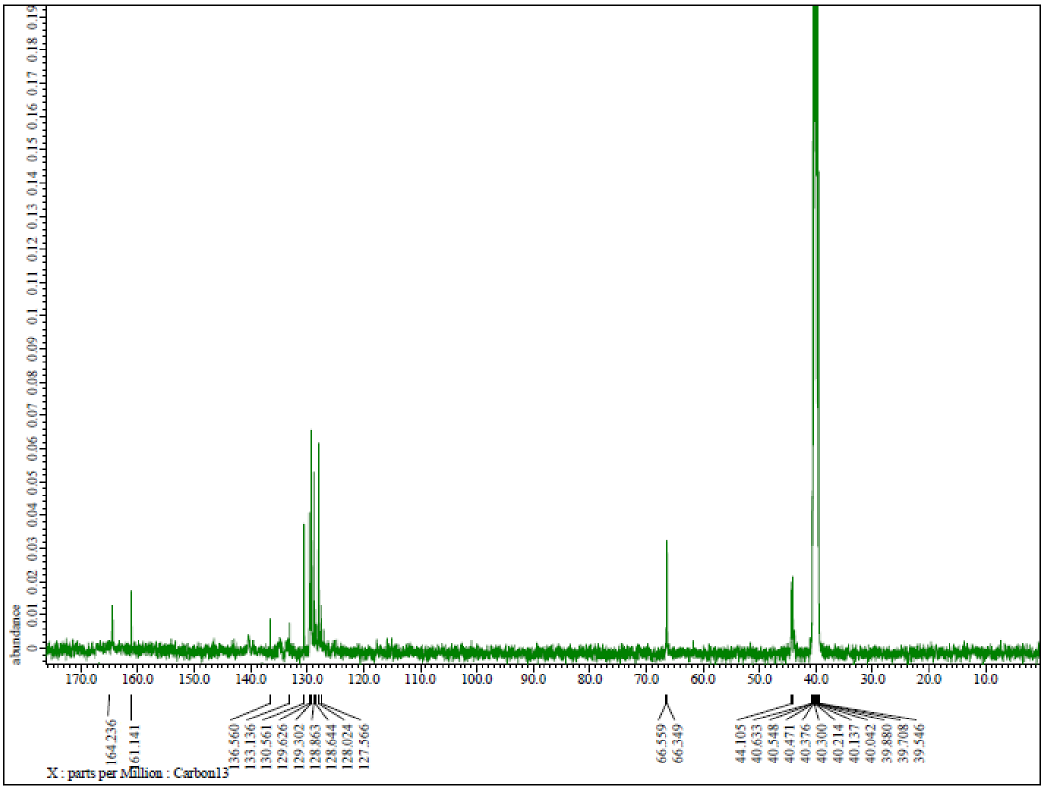

Figure S9: ^{1}H-NMR and ^{13}C-NMR for compound **4i**

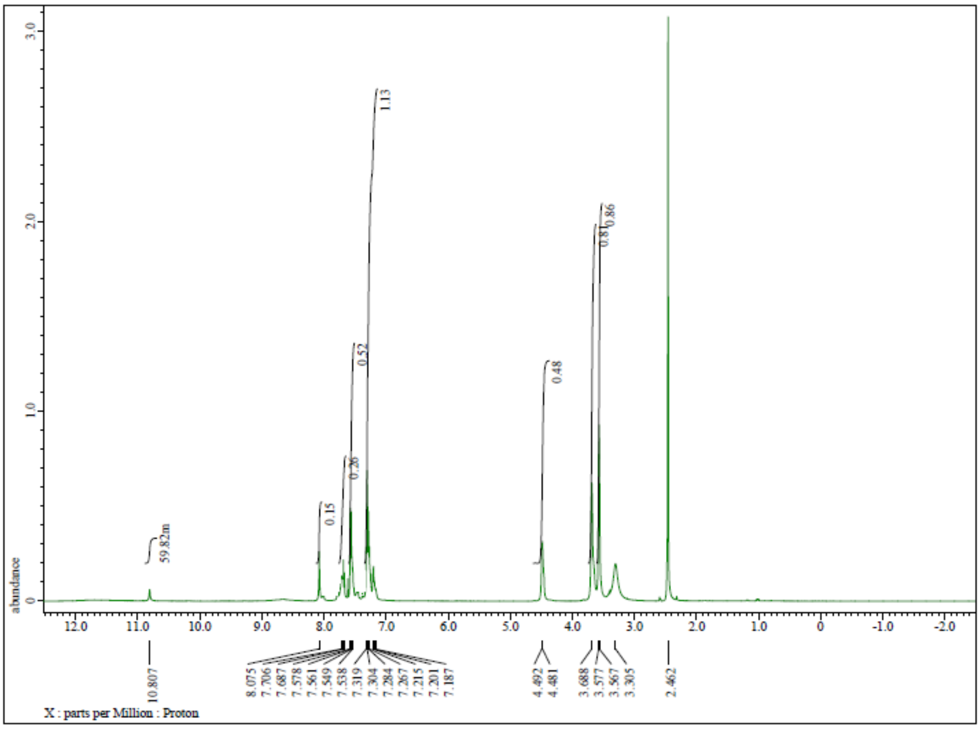

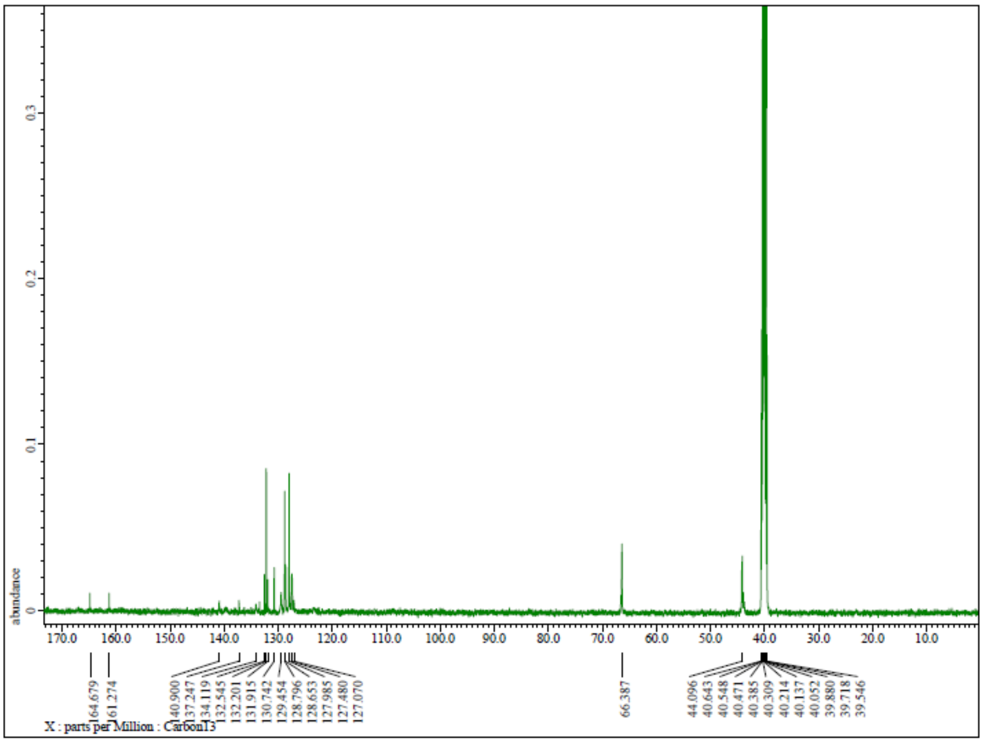

Figure S10: ^{1}H-NMR and ^{13}C-NMR for compound **4j**

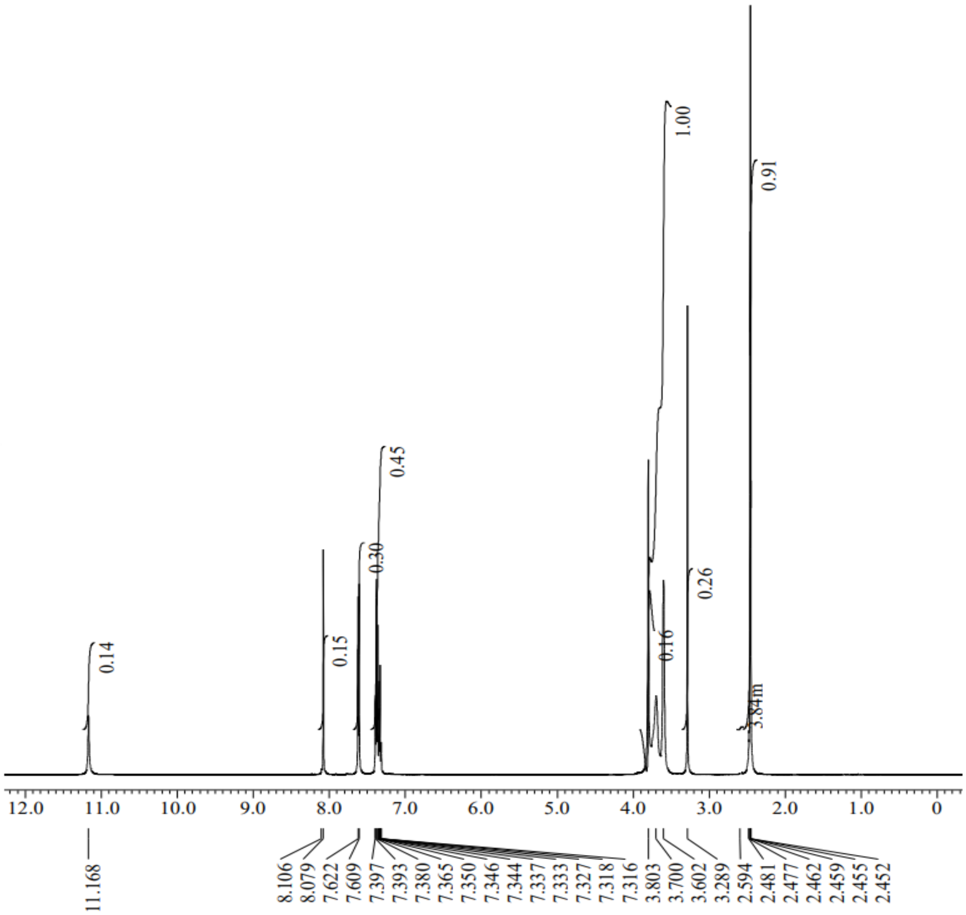

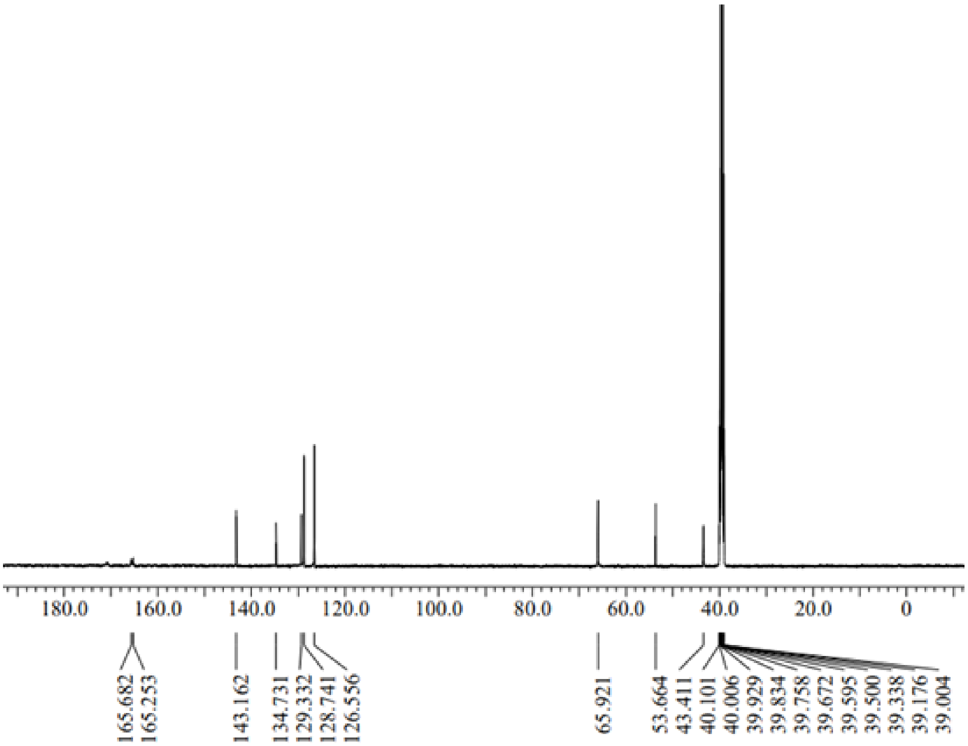

Figure S11: ^{1}H-NMR and ^{13}C-NMR for compound **4k**

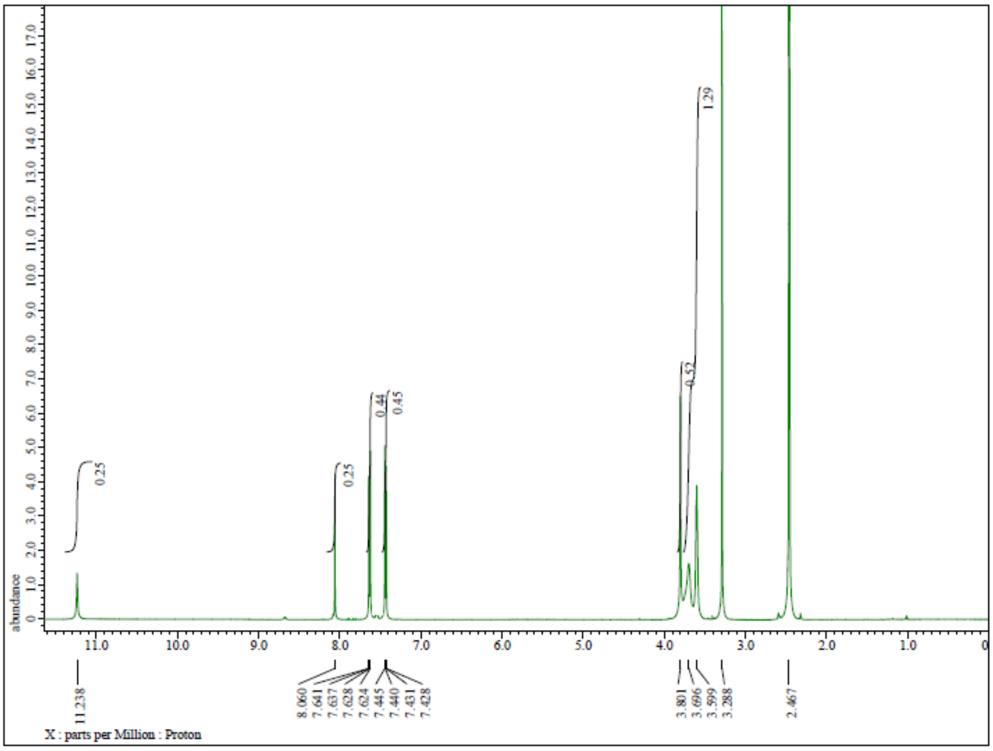

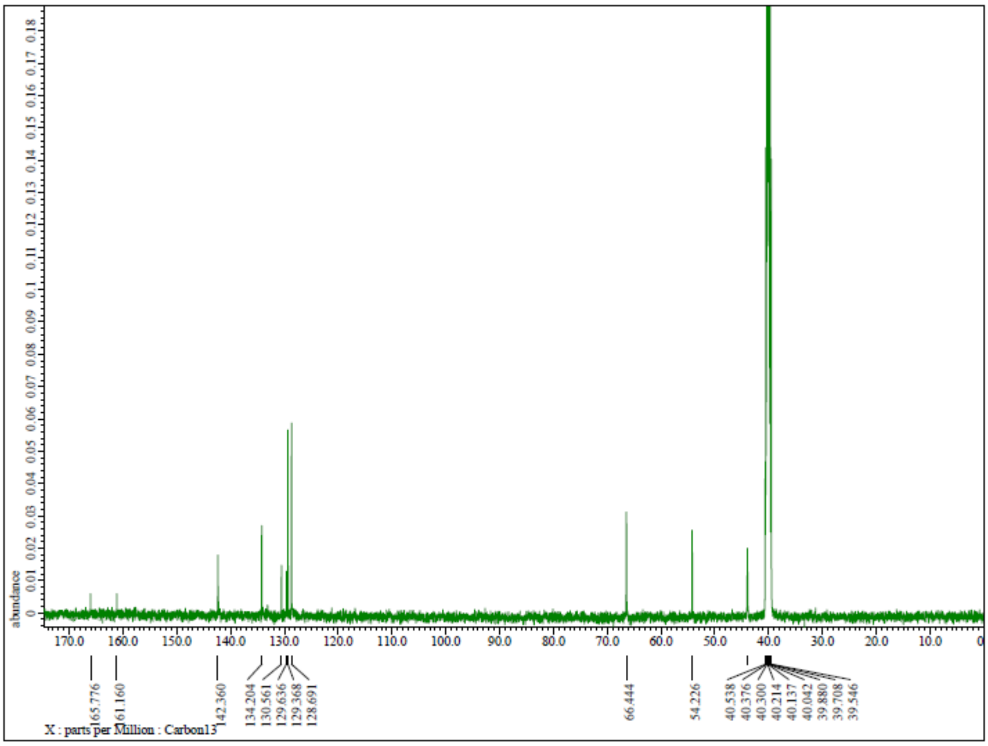

Figure S12: ^{1}H-NMR and ^{13}C-NMR for compound **4l**

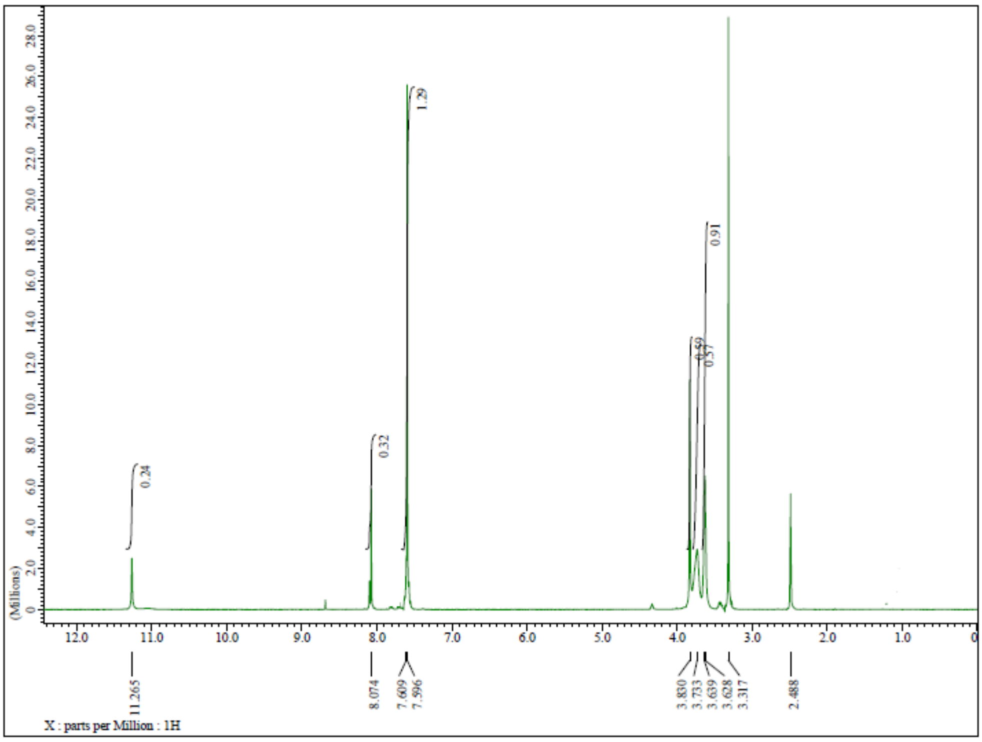

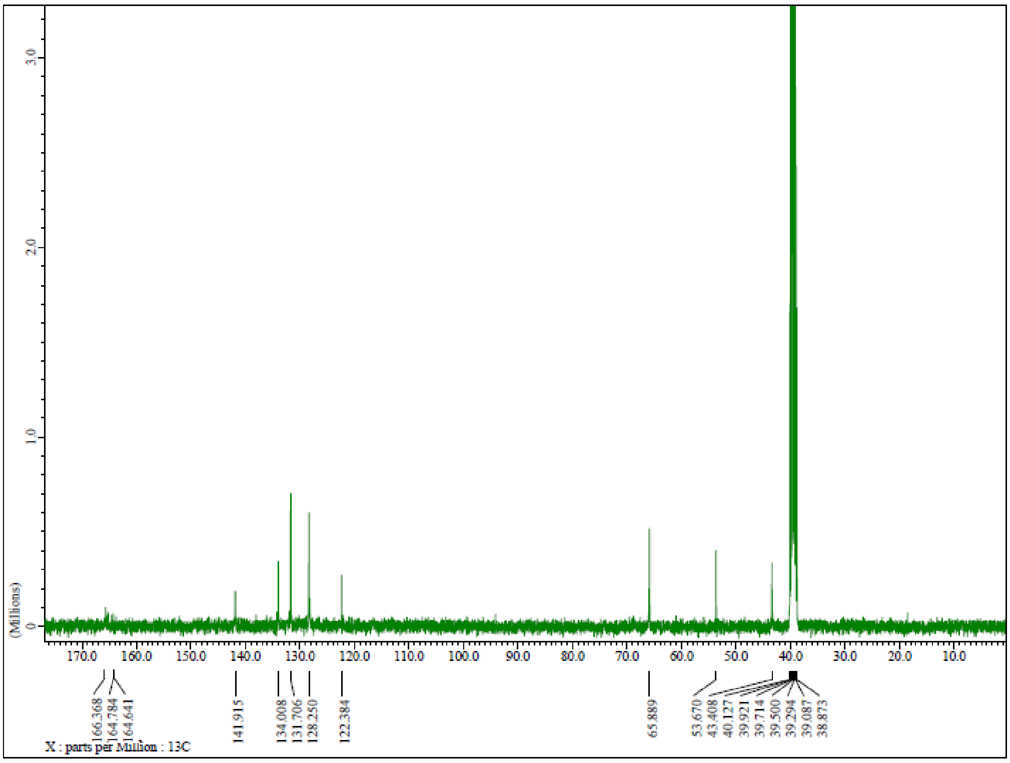

Figure S13: ^{1}H-NMR and ^{13}C-NMR for compound 4m

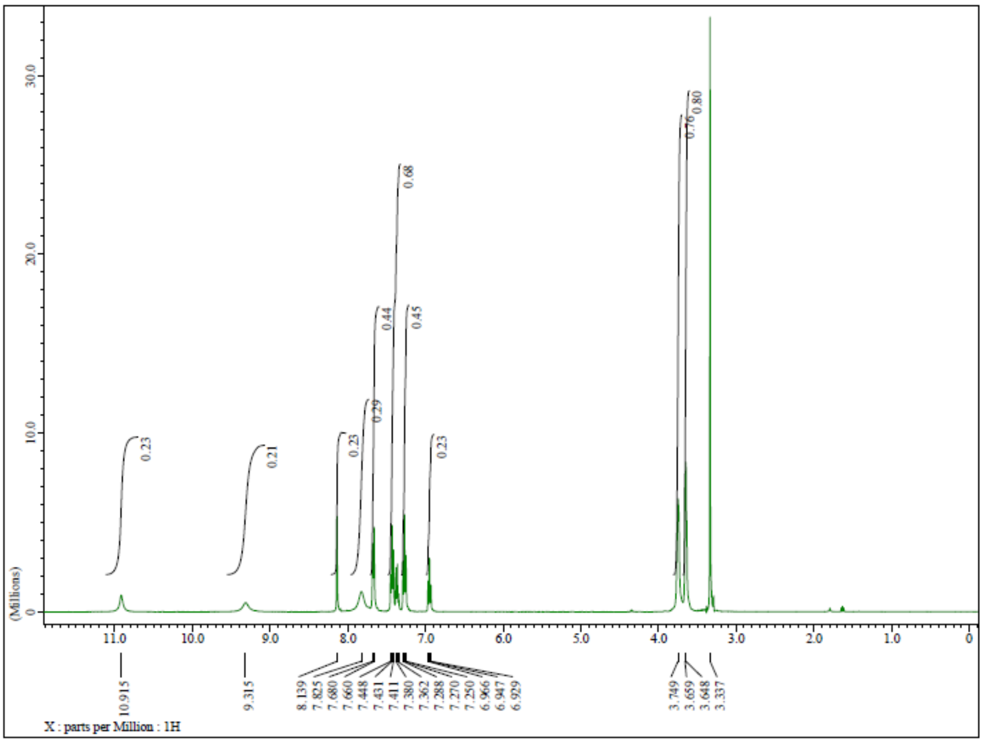

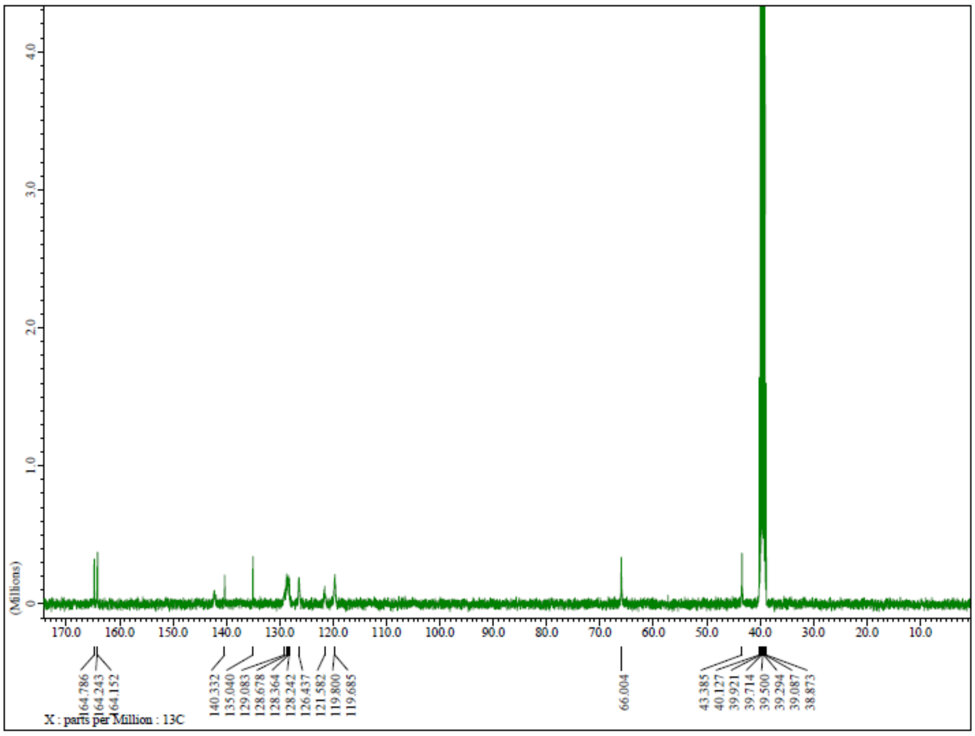

Figure S14: ^{1}H-NMR and ^{13}C-NMR for compound 4n

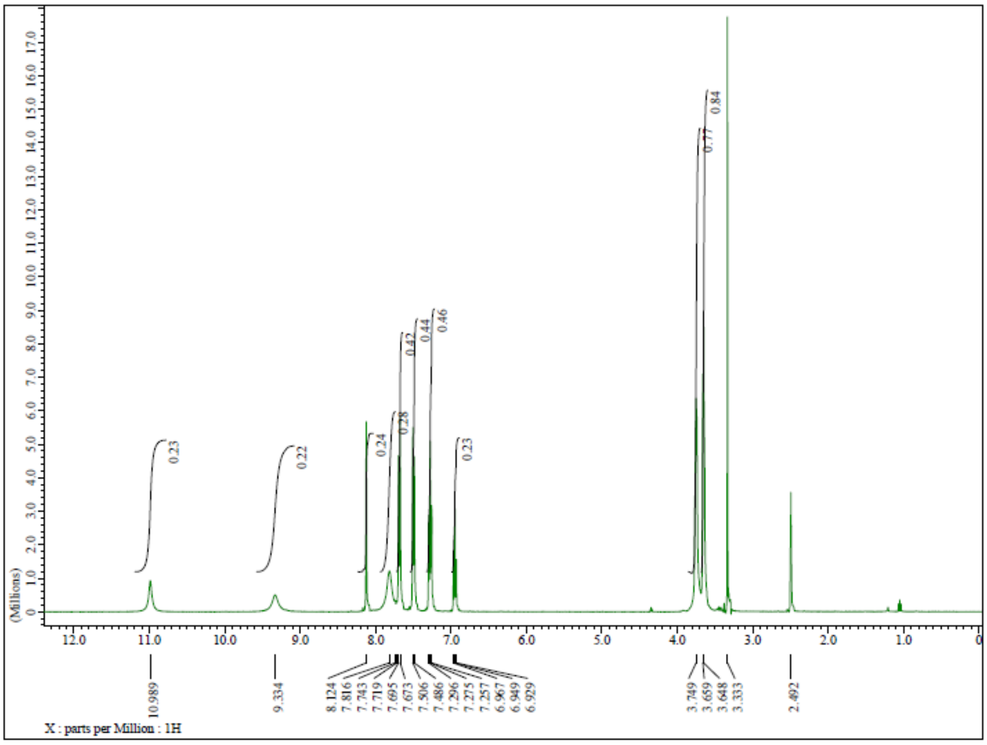

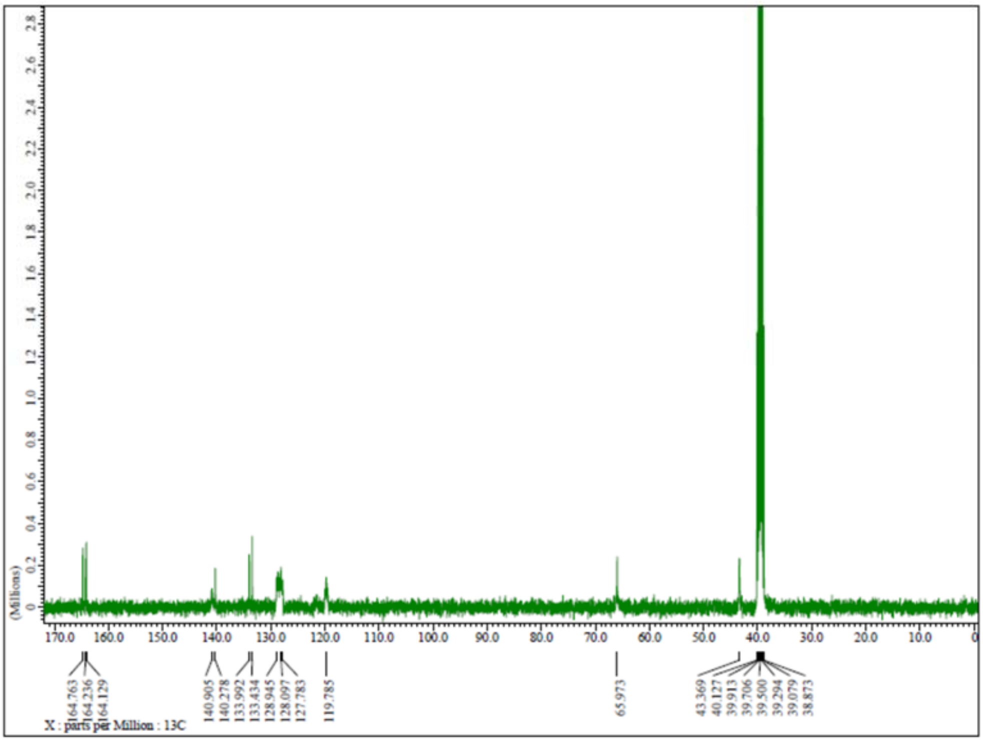

Figure S15: ^{1}H-NMR and ^{13}C-NMR for compound 4o

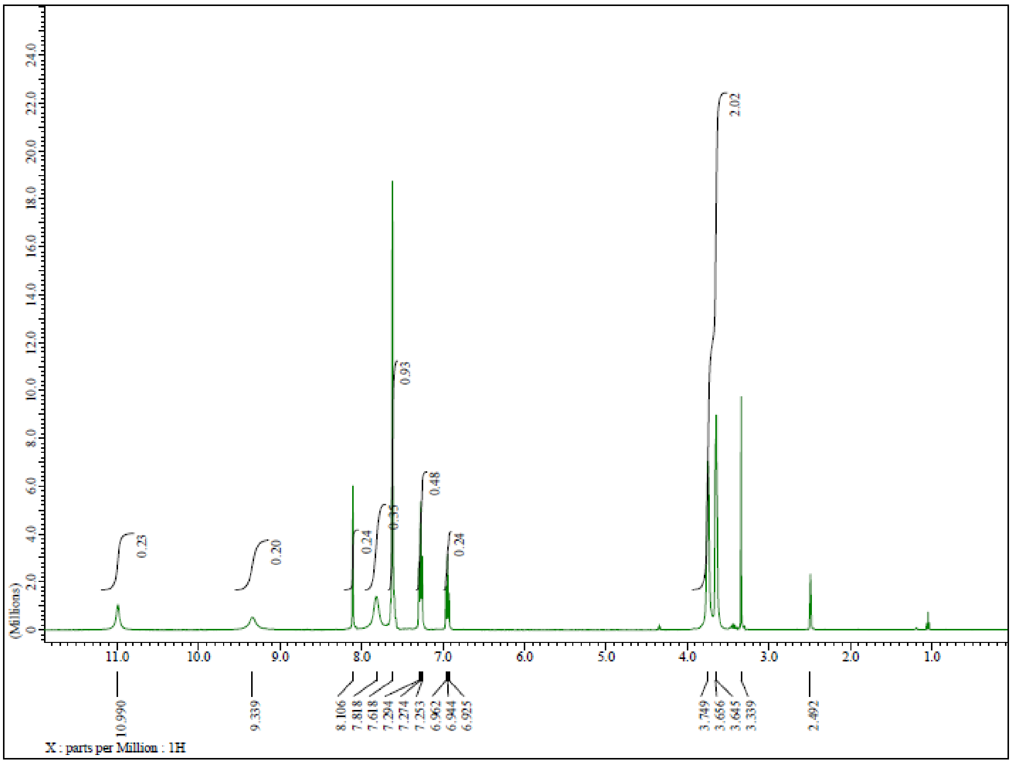

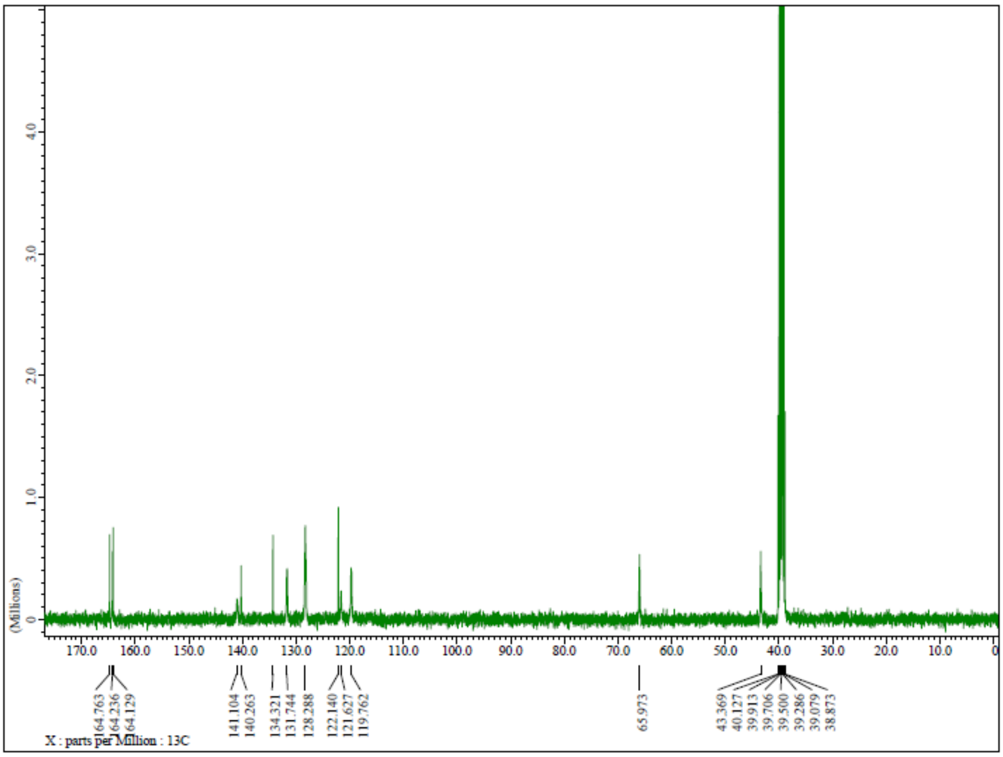

Figure S16: ^{1}H-NMR and ^{13}C-NMR for compound 4p

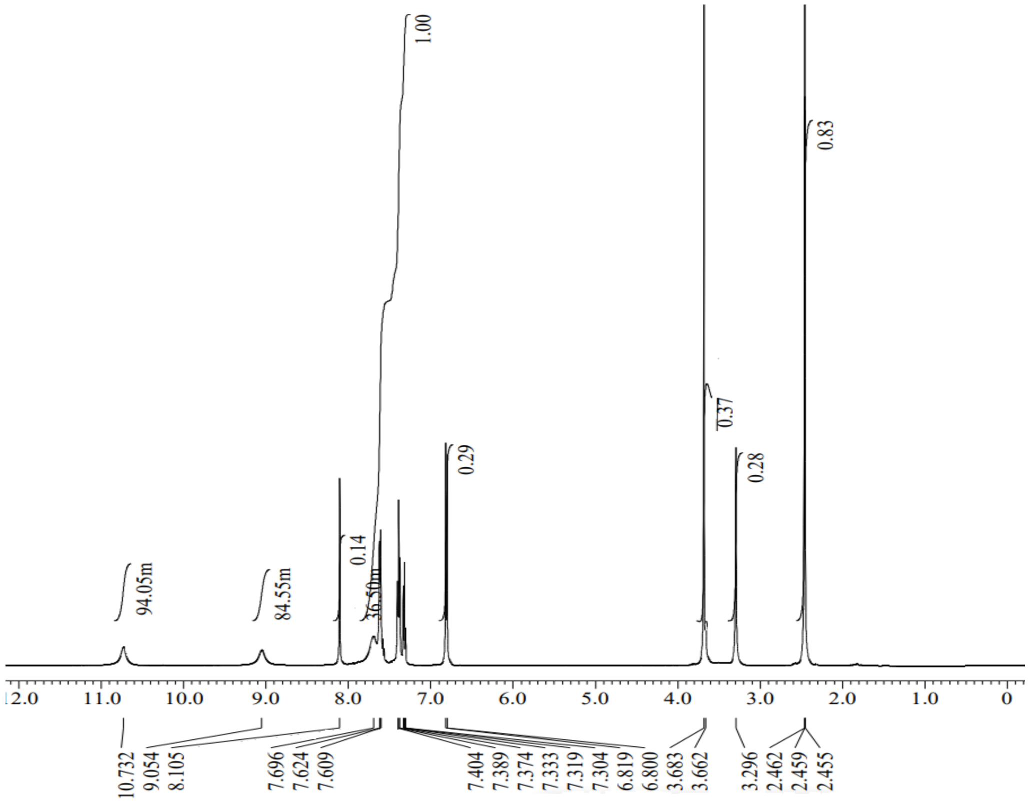

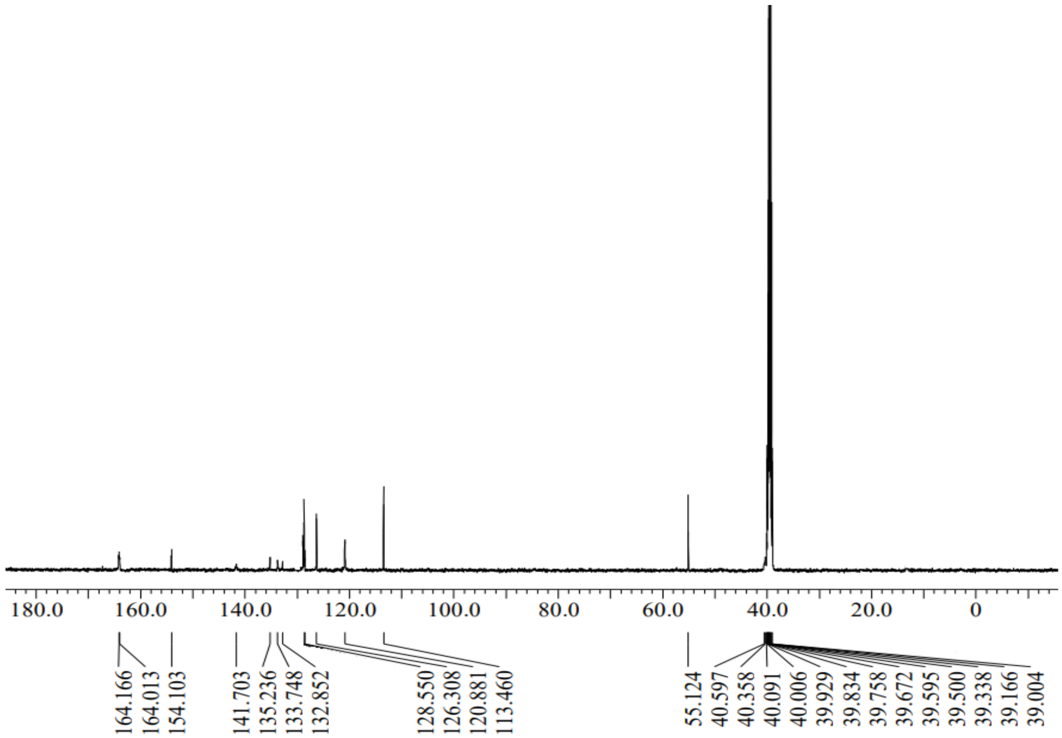

Figure S17: ^{1}H-NMR and ^{13}C-NMR for compound 4q

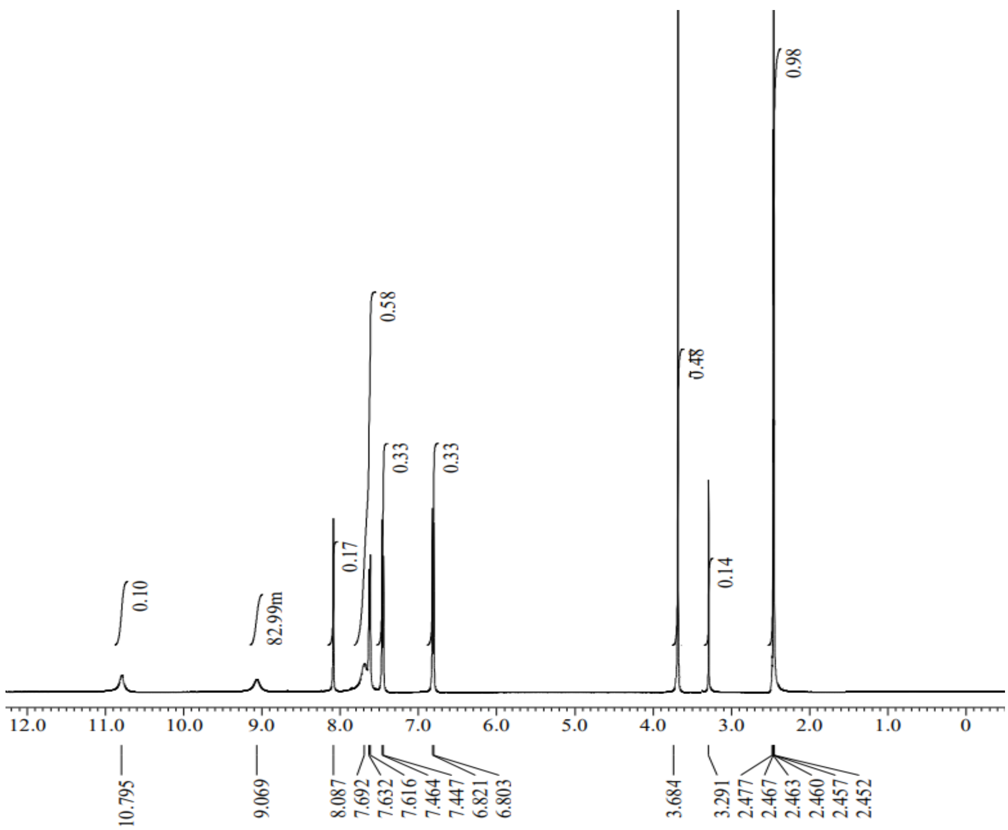

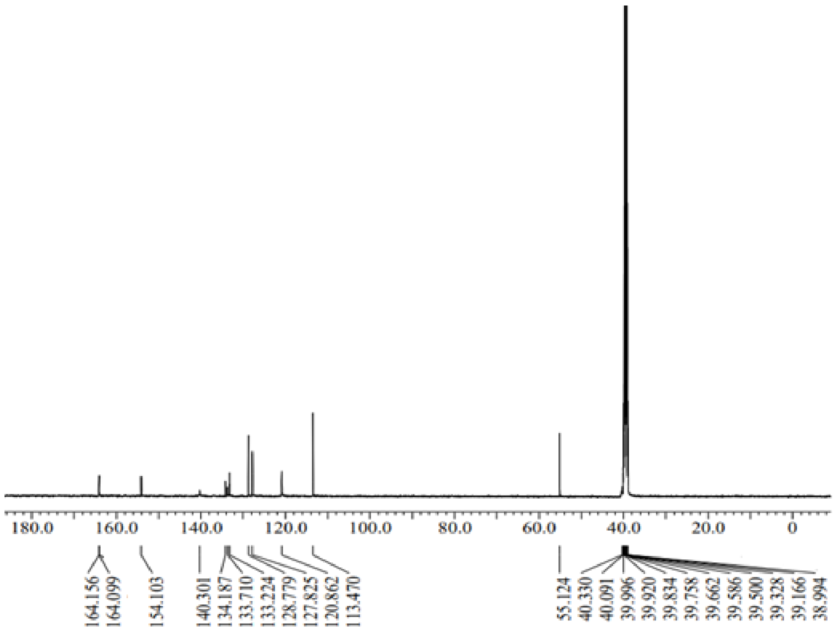

Figure S18: ^{1}H-NMR and ^{13}C-NMR for compound 4r

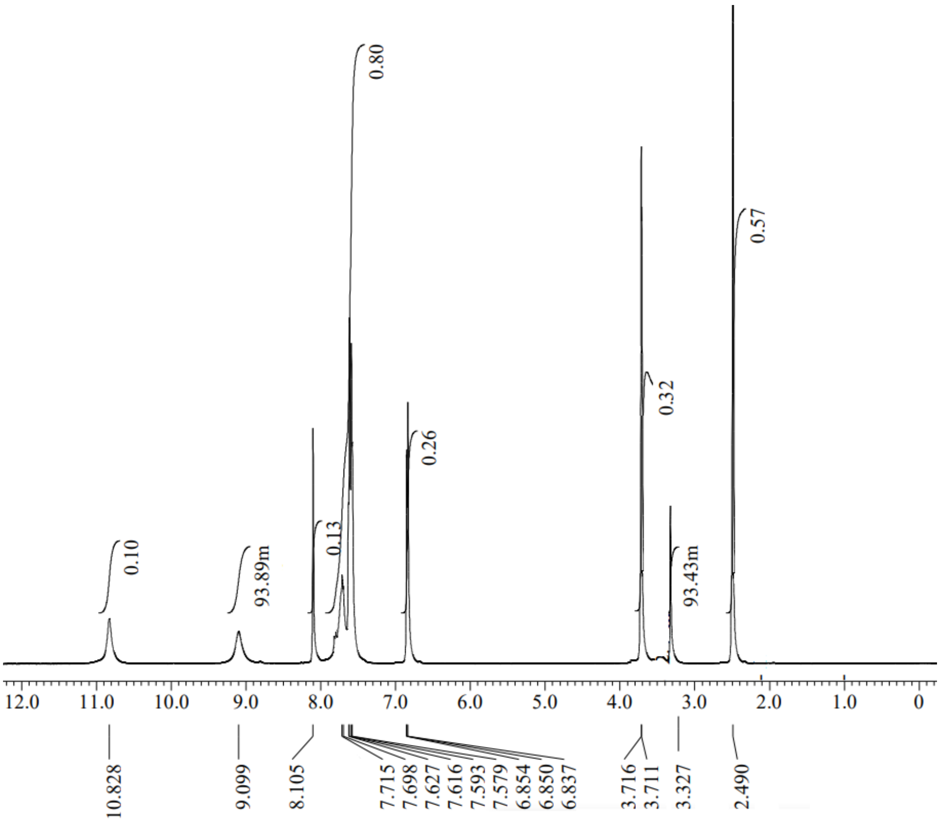

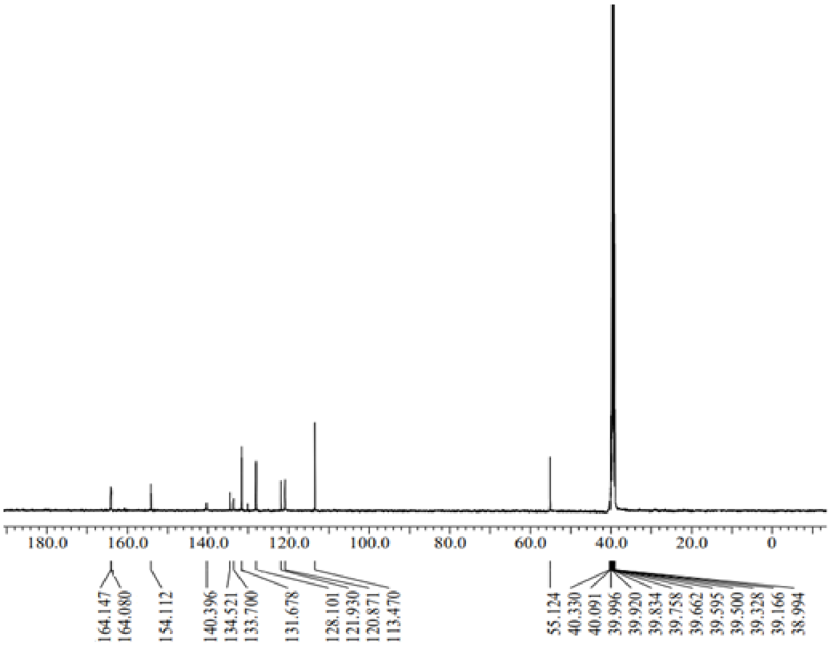

Supplement: Supplementary file 1 [file molecules-25-04065-s001.pdf]
